# The links between ecosystem multifunctionality and above- and belowground biodiversity are mediated by climate

**DOI:** 10.1038/ncomms9159

**Published:** 2015-09-02

**Authors:** Xin Jing, Nathan J. Sanders, Yu Shi, Haiyan Chu, Aimée T. Classen, Ke Zhao, Litong Chen, Yue Shi, Youxu Jiang, Jin-Sheng He

**Affiliations:** 1 Department of Ecology, College of Urban and Environmental Sciences, and Key Laboratory for Earth Surface Processes of the Ministry of Education, Peking University, Beijing 100871, China; 2 Center for Macroecology, Evolution and Climate, Natural History Museum of Denmark, University of Copenhagen, Copenhagen DK-2100, Denmark; 3 State Key Laboratory of Soil and Sustainable Agriculture, Institute of Soil Science, Chinese Academy of Sciences, Nanjing 210008, China; 4 Natural History Museum of Denmark, University of Copenhagen, DK-2100 Copenhagen, Denmark; 5 Key Laboratory of Adaptation and Evolution of Plateau Biota, Northwest Institute of Plateau Biology, Chinese Academy of Sciences, Xining 810008, China; 6 Institute of Botany, Chinese Academy of Sciences, Beijing 100093, China; 7 Institute of Forest Ecology, Chinese Academy of Forestry, Beijing 100091, China

## Abstract

Plant biodiversity is often correlated with ecosystem functioning in terrestrial ecosystems. However, we know little about the relative and combined effects of above- and belowground biodiversity on multiple ecosystem functions (for example, ecosystem multifunctionality, EMF) or how climate might mediate those relationships. Here we tease apart the effects of biotic and abiotic factors, both above- and belowground, on EMF on the Tibetan Plateau, China. We found that a suite of biotic and abiotic variables account for up to 86% of the variation in EMF, with the combined effects of above- and belowground biodiversity accounting for 45% of the variation in EMF. Our results have two important implications: first, including belowground biodiversity in models can improve the ability to explain and predict EMF. Second, regional-scale variation in climate, and perhaps climate change, can determine, or at least modify, the effects of biodiversity on EMF in natural ecosystems.

## Abstract (web summary)

Plant biodiversity often has a positive influence on ecosystem functioning. Here Jing *et al*. show that belowground diversity can also significantly impact ecosystem multifunctionality, but its relative effect varies by regional-scale climate variation.

## Graphical abstract

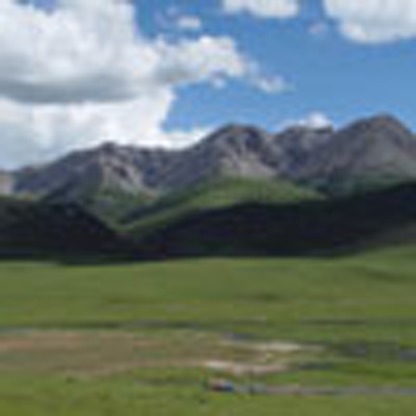

Ecologists have explored the links between biodiversity and ecosystem functioning for more than two decades^1^,^2^,^3^,^4^. Yet, most of our understanding of biodiversity–ecosystem function (BEF) relationships comes from experimental or observational investigations of how a single ecosystem function, usually aboveground net primary productivity, depends on plant biodiversity in small plots at single sites^2^,^4^,^5^ (but see refs 3, 6 for multisite comparisons). Clearly, ecosystems perform multiple functions simultaneously (ecosystem multifunctionality; EMF)^7^,^8^,^9^,^10^,^11^, and these functions can be mediated, if not controlled, by biodiversity in other components of ecosystems, such as the biodiversity of taxa belowground^11^,^12^,^13^,^14^,^15^,^16^,^17^. BEF relationships might also vary geographically, perhaps because of spatial or temporal variation in climate or other abiotic factors^3^,^5^,^9^,^18^.

Although above- and belowground communities and their interactions can shape multiple ecosystem functions, it is difficult to tease apart their relative effects^16^, perhaps because taxa operate at different spatial scales. For instance, belowground communities can be hyperdiverse over relatively small spatial scales^16^, and even Darwin recognized that these communities can be diverse and important^19^. However, while awareness of the immense biodiversity belowground is increasing, we are only beginning to elucidate the influence of belowground biodiversity on ecosystem functions^16^,^20^. Using integrative measures of biodiversity and multifunctionality should increase our ability to predict how biodiversity across taxa, both above- and belowground, shapes the suite of functions and services that ecosystems provide^10^,^21^.

While biodiversity can influence ecosystem function, climate can directly and indirectly affect it as well^9^,^22^ (see Supplementary Fig. 1). For example, climate directly influences ecosystem function by accelerating the activity and interactions among consumers, detritivores, decomposers and microbes^12^,^23^,^24^, and it indirectly affects ecosystem function by altering the composition of communities^16^. A growing number of experimental studies have crossed climate manipulations with biodiversity treatments to examine the interplay of these two factors on ecosystem functions^3^,^8^,^25^. Observational studies have taken advantage of natural climatic variation across multiple sites to explore how climate and aboveground (but rarely, if ever, belowground) biodiversity affect EMF. Together, these studies highlight the links among biodiversity and climate on EMF, and to some extent, geographic variation in the relationship between EMF and biodiversity^10^,^26^. Yet, there are few studies that explore geographic variation in how above- and belowground biodiversity, climate and ecosystem function are related. Thus, to understand the individual and combined influences of biodiversity and climate on EMF, a multifaceted approach where the links between biodiversity, both above- and belowground, and EMF across extensive climatic gradients is needed. Such an approach is critical for understanding BEF relationships, documenting whether these effects are large enough to rival the effects of the many other drivers of EMF, and scaling experimental results to real ecosystems at larger spatial scales^18^. Moreover, such an approach has important relevance for society: linking biodiversity above- and belowground to EMF across climatic gradients can better inform predictions about the interacting effects of changing climate and the loss and gain of species on the functioning of ecosystems^11^,^18^.

Here we examine how biodiversity above- (plant species richness) and belowground (for example, bacterial operational taxonomic units (OTUs), faunal richness, archaeal OTUs and arbuscular mycorrhiza (AM) fungal richness), and climate and soil factors (Supplementary Table 1) influenced EMF at 60 sites arrayed along an extensive climatic gradient in alpine grasslands on the Tibetan Plateau, China (Supplementary Fig. 2). We predicted that (1) soil and plant biodiversity would be positively correlated with EMF, but plant biodiversity would better predict EMF^9^,^11^; (2) the combined effects of above- and belowground biodiversity would be a stronger predictor of EMF than either above- or belowground biodiversity alone^7^,^11^; (3) climate and other factors that vary geographically would, at least partially, mediate the effects of above- and belowground biodiversity on EMF (Supplementary Fig. 2)^9^,^20^,^27^,^28^. Our results indicate that EMF is positively associated with the biodiversity of plant, soil bacteria and soil fauna, but not related to the biodiversity of soil archaea or AM fungi. Moreover, the combined effects of above- and belowground biodiversity explained more of the variation in EMF among sites than did either factor alone, and models that included climatic and soil factors as well as above- and belowground biodiversity accounted for up to 86% of the variation in EMF among sites.

## Results

### Relationships between biodiversity and EMF

**Figure 1:**
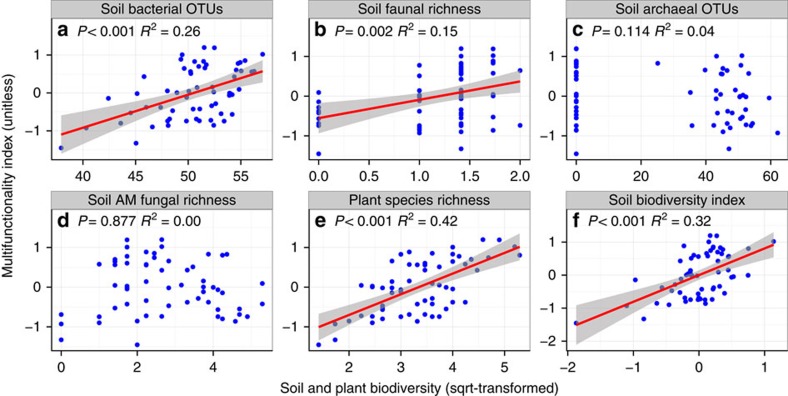
Relationships between below- and aboveground biodiversity and ecosystem multifunctionality. Relationships between soil bacterial (**a**) soil faunal (**b**) soil archaeal (**c**) soil AM fungal (**d**) plant (**e**) and soil (**f**) biodiversity and ecosystem multifunctionality (EMF). There were no significant linear relationships between EMF and soil archaeal biodiversity or soil AM fungal biodiversity. The red fitted lines are from OLS regression. Only significant fitted lines are displayed on the graphs. Shaded areas show 95% confidence interval of the fit.

To examine whether there was a significant linear relationship between biodiversity and each component of EMF (Supplementary Fig. 3–8), as well as whether there was a significant linear biodiversity–EMF relationship, we conducted ordinary least squares (OLS) regressions using site means (*n*=60) for each above- and belowground component of the ecosystem (Fig. 1). As predicted, the relationship between biodiversity and EMF varied among below- and aboveground components of the ecosystem. Biodiversity, both aboveground (plant species richness) and belowground (soil biodiversity index, see Estimating biodiversity in the Methods section), was positively correlated with EMF, but the belowground effect varied among components: some groups were positively related (bacteria and fauna), while others were not related (archaea and AM fungi) to EMF (Fig. 1a–d). Aboveground biodiversity (that is, plant species richness) explained more of the variation in EMF (42%) than did soil biodiversity (32%) (Fig. 1e,f).

### The effects of above- and belowground biodiversity on EMF

**Figure 2:**
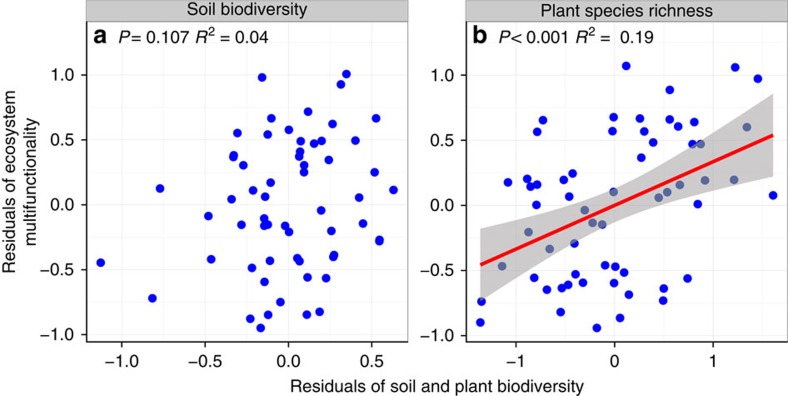
The relative effects of below- and aboveground biodiversity on ecosystem multifunctionality. The relative effects of soil biodiversity (**a**) and plant species richness (**b**) on ecosystem multifuncitonality (EMF).The red fitted line is from a partial linear regression. Only significant fitted lines are shown on the graphs. The results of variance partitioning for soil biodiversity and plant species richness are shown by the R^2^ on this graph. Shaded areas show 95% confidence interval of the fit.

Most previous BEF studies have considered only plant species richness, while largely ignoring biodiversity belowground. Here we examined the combined effects of above- and belowground biodiversity on EMF using general linear models (GLMs). We found that 45% of the variation in EMF was accounted for by above- and belowground biodiversity (Supplementary Table 2). Next, we fitted partial linear models to tease apart the relative effects of above- and belowground biodiversity on EMF (Fig. 2). Plant species richness accounted for 19% of the variation in EMF. Soil biodiversity, however, accounted for 4% of the variation in EMF, although this result was not statistically significant (*P*=0.107, partial linear regression). As predicted, the combined effects of above- and belowground biodiversity explained more of the variation (22%) in EMF than did either factor alone. Overall, plant species richness alone was the best single predictor of EMF (Figs 1e, 2).

### The effects of biodiversity and abiotic factors on EMF

To determine further whether the observed effects of above- and belowground biodiversity influenced EMF to the extent that abiotic factors did, we conducted regression as well as correlation analyses with mean annual precipitation (MAP), mean annual temperature (MAT), soil moisture, soil pH and soil CaCO_3_ as predictors in the models (Supplementary Figs 9 and 10). Then, we derived seven components from a principal component analysis (PCA) with the seven predictors mentioned above (Supplementary Fig. 11). The full model, with the seven components, accounted for 86% of the variation in EMF, and the best-fit model with two components (PC1 and PC4) accounted for 84% of the variation in EMF. These results indicate that the components of PC1 and PC4 were the strongest drivers of EMF (Supplementary Table 3). In addition, we found that PC1 represented MAP, soil moisture, soil pH, soil CaCO_3_, aboveground biodiversity and belowground biodiversity. PC4 represented MAT, soil moisture and soil CaCO_3_ (Supplementary Fig. 11).

Next, we fitted GLMs to investigate the biotic and abiotic effects on EMF (Table 1). We found that soil moisture was the most important driver, accounting for 65% of the variation in EMF (Supplementary Fig. 9). In total, 86% of the variation in EMF was accounted for by a model that included soil pH, aboveground biodiversity, belowground biodiversity and MAT (Table 1). MAP and soil CaCO_3_ had no influence on the model predictions. However, we found that MAP explained 43%, soil pH explained 30% and soil CaCO_3_ explained 33% of the variation in EMF (Supplementary Fig. 9). MAP and soil moisture were positively, and soil pH and soil CaCO_3_ were negatively, correlated with EMF. Somewhat surprisingly, we did not detect any relationship between temperature and EMF (Supplementary Fig. 9).

**Figure 3:**
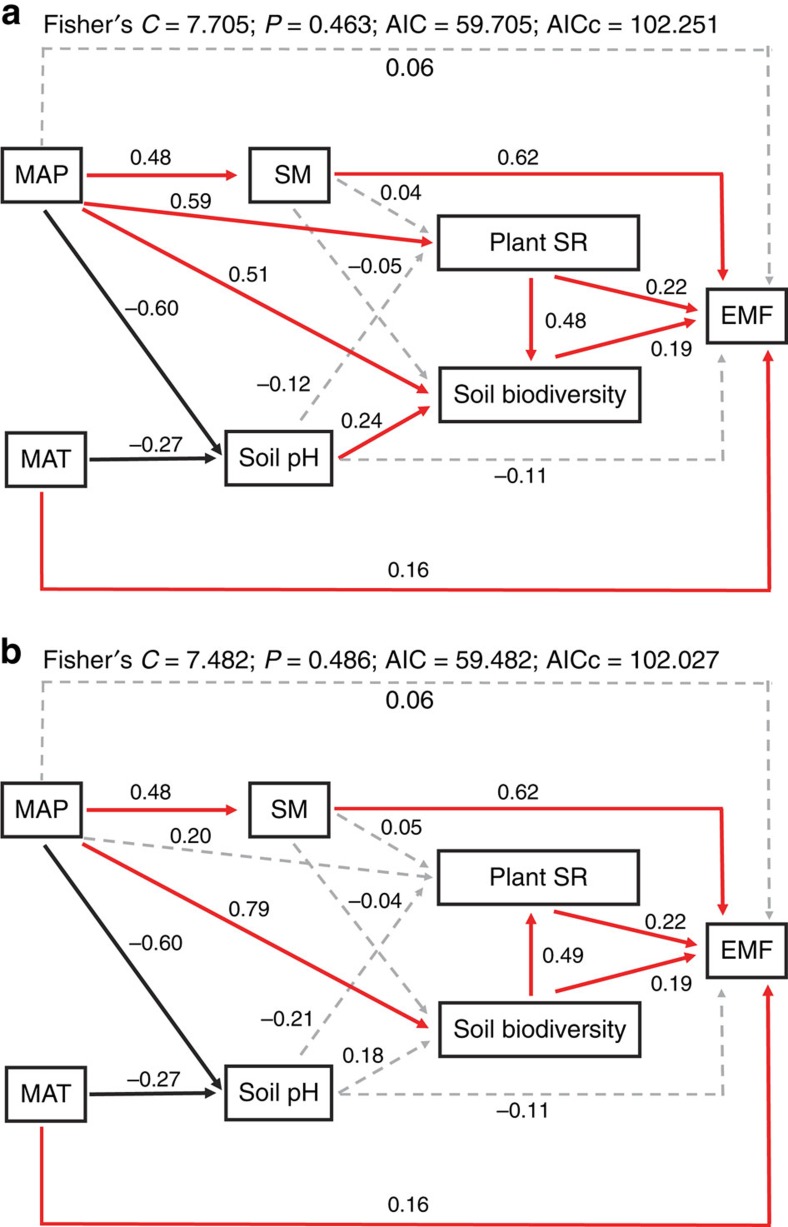
Structural equation models of climate, soil and biodiversity as predictors of ecosystem multifuncitonality (EMF). Solid red arrows represent positive paths (*P*<0.05, piecewise s.e.m.), solid black arrows represent negative paths (*P*<0.05, piecewise s.e.m.) and dotted grey arrows represent non-significant paths (*P*>0.05, piecewise s.e.m.). We report the path coefficients as standardized effect sizes. Overall fit of piecewise s.e.m. was evaluated using Shipley's test of d-separation: Fisher's *C* statistic (if *P*>0.05, then no paths are missing and the model is a good fit) and Akaike information criterion (AIC). MAP, mean annual precipitation; MAT, mean annual temperature; SM, soil moisture, plant SR, plant species richness.

Finally, we fitted a piecewise structural equation model (SEM) to infer the direct and indirect effects of climate (MAP and MAT), soil properties (soil moisture and soil pH), and above- and belowground biodiversity on EMF. Overall, two models with Fisher's *C* statistic *P*>0.05 and ΔAICc=0.224 were selected (Fig. 3). The SEM demonstrated that the influence of precipitation on EMF was mediated through soil moisture, aboveground biodiversity, belowground biodiversity and soil pH. Temperature directly (*β*=0.16, standardized coefficient), and indirectly through soil pH (*β*=−0.27, standardized coefficient), impacted EMF. Soil moisture was the most significant parameter (*β*=0.62, standardized coefficient) influencing EMF. Aboveground and belowground biodiversity were interactively and approximately equal in magnitude in their influence on each other as well as on EMF (Fig. 3). The strongest relationship observed in the SEM analysis was between MAP and belowground biodiversity (*β*=0.79, standardized coefficient; Fig. 3b). There was also a weaker and negative relationship between soil pH and EMF (*β*=−0.11, standardized coefficient) (Fig. 3).

## Discussion

Though debate exists about the mechanisms by which biodiversity is related to ecosystem function, numerous studies have demonstrated that aboveground biodiversity (that is, plant species richness) tends to be positively correlated with ecosystem function^26^,^29^. Recent studies have expanded these analyses and explored the relationships among biodiversity and multiple ecosystem functions. Similar to the classical BEF work, these studies tend to find that aboveground biodiversity is generally positively related to EMF^9^,^11^,^21^,^30^. Our study, across an extensive climatic gradient on the Tibetan Plateau, compliments the growing EMF literature by demonstrating that biodiversity and multiple ecosystem functions were positively correlated. However, our results are unique in that the combined effects of above- and belowground biodiversity accounted for a relatively large fraction (45%) of the observed variation in EMF across sites. Moreover, these relationships were strongly modulated by climatic variation. The best-fitting models accounted for 86% of the variation in EMF and included soil moisture, soil pH, above- and belowground biodiversity as well as climatic variables (MAT). Thus, our results indicate that the abiotic environment dictates, or at least modifies, the effects of biodiversity on the functioning of ecosystems.

Combining belowground biodiversity with measures of aboveground biodiversity increased the predictive power of biodiversity on EMF (Supplementary Table 2; Fig. 3). Plant species richness was always positively related to EMF. Belowground biodiversity alone, however, accounted for only 4% of the variation in EMF (Fig. 2). There are several explanations for the differences in above- and belowground impacts on EMF. First, the influence of belowground biodiversity on EMF varied among taxa (Fig. 1). Soil bacterial and soil faunal biodiversity were positively correlated with EMF, as was plant species richness, but diversities of archaea and AM fungi were not correlated with EMF. Thus, the effects of biodiversity of multi-trophic organisms and ecosystem functions^31^,^32^ may mediate the positive effects of plant, bacterial and faunal biodiversity on EMF^9^,^33^,^34^. Climatic and other environmental differences among sites, such as MAP, may also be key drivers in shaping the positive relationship between biodiversity and EMF as well as the differences between above- and belowground communities^26^. Such a mechanism was supported by the SEMs (Fig. 3) where the influence of precipitation on EMF was mediated through soil moisture, soil pH, plant species richness and soil biodiversity. Finally, soil bacteria and soil fauna are relatively broad taxonomic groupings with diverse associated traits and functions, while archaea and AM fungi are more narrow taxonomic groupings with a more limited suite of functions. Thus, increasing biodiversity of broad taxonomic groups of free-living soil organisms, such as bacteria, should increase the diversity of substrates that are decomposed in the soil and returned to the plant community for uptake^35^, which should increase EMF.

The relationship between EMF and the biodiversity of AM fungi and archaea may reflect differences in the life histories of these taxa. AM fungi are closely tied to plants and often provide limiting nutrients such as phosphorus to plants^36^. In the current study, there were no effects of AM fungal biodiversity on EMF. However, when we considered the effects of AM fungal biodiversity on single ecosystem functions, we found that some relationships were negative, some were positive and some were non-significant (Supplementary Fig. 4). For example, AM fungal biodiversity was negatively correlated with soil phosphorus concentration, which could offset the positive effects of AM fungal biodiversity on root biomass and phosphorus content in aboveground biomass. Thus, the average of multiple functions encompassed in the index of EMF might not be a good metric to reflect the multiple and often interacting functions performed by AM fungi or the complex interactions between plant hosts and AM fungi. Similar to AMF, archaea showed a different pattern with EMF than did soil bacteria; however, it is premature to speculate about their influence on EMF given that their potential ecological impact and response to climate is still being elucidated^36^,^37^.

Our results underscore the obvious fact that the abiotic environment influences EMF. At least on the Tibetan Plateau (and probably elsewhere), abiotic factors, such as precipitation and soil moisture, are key drivers shaping the biodiversity–EMF relationship (Fig. 3; Table 1; Supplementary Fig. 9). While our estimates of soil moisture come from a single measurement and are not integrated across the entire season (see Supplementary Note 1 for an extensive discussion of soil moisture), they were collected at the same time and were comparable to one another among the sites. Further, in alpine grassland ecosystems, higher water availability is generally associated with higher precipitation, higher aboveground net primary productivity and higher nutrient availability^26^,^38^,^39^, which could facilitate resource use by plants^8^,^40^ and ultimately support more species.

In contrast to soil moisture, MAT generally had no effect on among-site variation in ecosystem functions when we considered it in isolation of other potential factors (Supplementary Fig. 9a). This pattern may emerge because, in this ecosystem, diurnal variation in temperature is large and often approximates seasonal variation^41^, suggesting that the communities are adapted to extensive variation in temperature. Thus, our results contrast with similar work from dryland ecosystems^9^, and suggest that an increase in temperature will not affect EMF in these alpine grassland ecosystems. However, in combination with the other biotic (biodiversity both above- and belowground) and abiotic factors (MAP, soil moisture and soil pH) that were included in the GLM and SEM, MAT significantly increased the explanatory power of the models. Therefore, other environmental factors may covary with the temperature–EMF relationships in broad-scale studies across climates, soil types and plant communities. Again, teasing apart why temperature is related to EMF in some ecosystems but not others is an important challenge, given ongoing increases in global temperatures.

Soil CaCO_3_ was highly correlated with soil pH, and was a better predictor of EMF than was soil pH, yet soil pH emerged as an important driver of EMF. Grassland soils across northern China, including those on the Tibetan Plateau, have experienced significant acidification since the 1980s (ref. 42). Soil acidification should inhibit decomposition of soil organic C, which might enhance the ability of alpine grassland ecosystems to perform multiple functions. Soil pH has been highlighted in other studies as being an important predictor of belowground biodiversity^43^.

Given that soils sustain life aboveground, a better understanding of the relative and combined effects of above- and belowground biodiversity is needed to predict the potential consequence of biodiversity loss for the future maintenance of ecosystem functions and services^20^. We note, however, that our approach to characterizing biodiversity belowground provides only a rough estimate of the actual belowground biodiversity because the sequencing methods we used did not detect all taxa (Supplementary Fig. 12). Hence, while unlikely, the relative effect of soil biodiversity on EMF may have been different had we used another suite of molecular methods to characterize soil biodiversity. Future studies linking above- and belowground biodiversity to EMF should use higher resolution sequencing profiling methods as well as techniques that focus on the active community to characterize the active component of belowground biodiversity. Despite this caveat, our work nevertheless demonstrates that abiotic factors (climate and soil) mediate the effects of above- and belowground biodiversity on EMF, which will depend strongly on the climatic gradients considered.

Belowground biodiversity, as well as biodiversity aboveground, clearly influence EMF. Our works build on a growing body of work suggesting that the correlation between biodiversity and EMF may be a general pattern across natural ecosystems^9^. However, our work also demonstrates that the relationship between components of biodiversity, here above- and belowground components of ecosystems, and EMF can be positively or not related. Moreover, biodiversity–EMF relationships may be strongly mediated by climate and be contingent on the environmental constraints of different ecosystems. Obviously, experimental work would help disentangle these factors further, and such experiments are a clear next step in EMF–biodiversity research globally. As climates change and species are lost and gained from ecosystems, predicting how ecosystems will function in the future will require experiments and observations that link biodiversity above- and belowground to EMF.

## Methods

### Sampling

We sampled plant and soil communities at 60 study sites over an extensive area (>1,000,000 km^2^) in the northeastern and central Tibetan Plateau in Qinghai Province and Tibetan Autonomous Region, China (Supplementary Fig. 2) during the peak-growing season (July–August) of 2011. Our survey captured a substantial range of the vegetation types, soil classes and climatic conditions found in the alpine grasslands on the Tibetan Plateau. The sites represent the three main vegetation types: alpine meadow, alpine steppe and desert steppe, and the 11 soil types (Genetic Soil Classification of China^44^): brown pedocals, castanozems, chernozems, cold calcic soils, dark felty soils, felty soils, frigid calcic soils, frigid frozen soils, grey–brown desert soils, grey-cinnamon soils and meadow soils on the Plateau. We selected sites to minimize the potential effects of grazers and other disturbances on soil and plant community structure. For each of the sites, we compiled MAT and MAP from the National Meteorological Bureau of China database. Data were compiled by interpolating data of monthly mean temperature and monthly precipitation records (1951–2010) from 716 climate stations across China (http://cdc.cma.gov.cn). The sites ranged in elevation from 2,918 to 5,228 m (mean, 4,064 m), in MAT from −5.2 to 4.7 °C (mean, −0.17 °C), and in MAP from 66 to 560 mm (mean, 365 mm) (Supplementary Table 4).

We collected 180 soil samples (0–5 cm; 3 samples per site) from the 60 sites. At each site, we established a 100-m transect and randomly placed three plots (1 × 1 m^2^) on each transect, with the stipulation that the plots were at least 40-m apart. Within each plot, 5–7 soil cores (5 cm in diameter) were collected, bulked, and homogenized in the field. Soil samples for total soil carbon, nitrogen, phosphorus and CaCO_3_ analysis were air-dried, sieved (2-mm mesh) and ground to a fine powder using a ball mill. Soil samples for molecular and physiochemical (soil moisture, soil available nitrogen and soil pH) analysis were packed in polyethylene bags, immediately stored in portable refrigerator powered with a car battery and then stored in the lab at −20 °C until processing^45^. Subsamples were in the freezer for no >1 week before measurements.

### Physiochemical data measurements

Total soil carbon and nitrogen were determined by combustion on a CHN elemental analyzer (2400 II CHN elemental analyzer, PerkinElmer, Boston, MA, USA). Total soil phosphorus was determined by the molybdenum blue method with a ultraviolet–visible spectrophotometer (UV-2550, Shimadzu, Kyoto, Japan). Soil CaCO_3_ was analysed volumetrically on ground subsamples using a Calcimeter (Eijkelkamp, Netherland). Soil organic carbon was calculated as the difference between total soil carbon and carbon bound in soil CaCO_3_. The density of soil organic carbon, soil nitrogen, soil phosphorus and soil CaCO_3_ were calculated in the top 0–5 cm. Soil moisture was measured gravimetrically after ∼10-h desiccation at 105 °C. Soil available nitrogen (sum of ammonium, nitrate and dissolved organic nitrogen) were determined using a TOC-TN analyzer (Shimadzu, Kyoto, Japan). Soil pH was determined in a 1:5 ratio of fresh soil to deionised water slurry on a pH meter (Thermo 0rion-868).

### DNA extraction and pyrosequencing

The biodiversity of soil bacteria, AM fungi and archaea were assessed using a suite of molecular techniques. The nucleic acids from each plot (*n*=180) were extracted from 0.5 g of soil using a FastDNA Spin kit (Bio 101, Carlsbad, CA, USA), according to the manufacturer's instructions, and then stored at −40 °C. Extracted DNA was diluted to ∼25 ng μl^−1^ with distilled water and stored at −20 °C until use. For bacterial, archaeal and AM fungal amplification 2 μm diluted DNA samples from each plot were used as a template.

To assess bacterial communities, we amplified the V4–V5 hypervariable regions of bacterial 16S ribosomal RNAs (rRNAs; *Escherichia coli* positions 515–907) using the primer set: F515 (ref. 46): 5′-GTGCCAGCMGCCGCGG-3′ with the Roche 454 ‘A' pyrosequencing adapter and a unique 7-bp barcode sequence, and primer R907 (ref. 47): 5′-CCGTCAATTCMTTTRAGTTT-3′ with the Roche 454 `B' sequencing adapter at the 5′-end of each primer. We conducted PCR amplification with 25-μl 2 × premix (TaKaRa), 0.5-μl 20 mM each forward and reverse primer and 50 ng of DNA, and the volume was completed to 50 μl with double-distilled water. Each sample was amplified in triplicate with the 50-μl reaction under the following conditions: 30 cycles of denaturation at 94 °C for 30 s, annealing at 55 °C for 30 s and extension at 72 °C for 30 s; with a final extension at 72 °C for 10 min^43^. PCR products from each sample were pooled together, purified with an agarose gel DNA purification kit (TaKaRa), quantified using a NanoDrop ND-1000 spectrophotometer (Thermo Scientific, USA), then combined in equimolar ratios in a single tube and run on a Roche FLX454 pyrosequencing machine (Roche Diagnostics Corp., Branford, CT, USA), producing reads from the forward direction F515.

To assess archaeal communities, the V3–V5 hypervariable regions of archaeal 16S rRNA^48^ were amplified using the primer set: Arch344F: 5′-ACGGGGYGCAGCAGGCGCGA-3′ with the Roche 454 ‘A' pyrosequencing adapter and a unique 7-bp barcode sequence, and primer Arch915R: 5′-GTGCTCCCCCGCCAATTCCT-3′ with the Roche 454 ‘B' sequencing adapter at the 5′-end of each primer. We conducted PCR amplification with 25-μl 2 × premix (TaKaRa), 0.5-μl 20 mM each forward and reverse primer and 50 ng of DNA, and the volume was completed to 50-μl with double-distilled water. Each sample was amplified in triplicate with the 50-μl reaction under the following conditions: 94 °C for 5 min, 10 cycles of touchdown PCR (denaturation at 94 °C for 30 s, annealing for 30 s with a 0.5 °C per cycle decrement at 61 °C above the respective annealing temperatures and extension at 72 °C for 1 min), followed by 25 cycles of regular PCR (94 °C for 30 s, 30 s at the respective annealing temperature, 72 °C for 1 min and a final extension step for 7 min at 72 °C)^48^. PCR products from each sample were pooled together and purified with an Agarose Gel DNA purification kit (TaKaRa), quantified using a NanoDrop ND-1000 spectrophotometer (Thermo Scientific, USA), then combined in equimolar ratios in a single tube and run on the Roche FLX454 pyrosequencing machine, producing reads from the forward direction Arch344F.

To assess AM fungal communities, the 18S rRNA gene fragment for the 454 GS-FLX pyrosequencing platform was amplified using the primer set: AMV4.5NF (5′-AAGCTCGTAGTTGAATTTCG-3′) with the Roche 454 ‘A' pyrosequencing adapter and a unique 7-bp barcode, and primer AMDGR (5′-CCCAACTATCCCTATTAATCAT-3′) with the Roche 454 ‘B' sequencing adapter at the 5′-end of each primer^49^. We conducted PCR amplification with 25-μl 2 × premix (TaKaRa), 0.5-μl 20 mM each forward and reverse primer and 50 ng of DNA, and the volume was completed to 50-μl with double-distilled water. Each sample was amplified in triplicate with the 50- μl reaction mixtures under the following conditions: 30 cycles of denaturation at 94 °C for 30 s, annealing at 58 °C for 30 s and extension at 72 °C for 30 s; with a final extension at 72 °C for 10 min^50^. PCR products from each sample were pooled together, purified with a Agarose Gel DNA purification kit (TaKaRa), quantified using a NanoDrop ND-1000 spectrophotometer (Thermo Scientific, USA) and then combined in equimolar ratios in a single tube. 454-sequencing was performed by BGI (Shenzhen, China) on a Roche FLX454 pyrosequencing machine, producing reads from the forward direction AMV4.5NF.

### Pyrosequencing data analyses

We processed and analysed bacterial and archaeal biodiversity data as described by Chu *et al*.^45^ and Hamady *et al*.^51^ with the Quantitative Insights into Microbial Ecology pipeline (http://qiime.sourceforge.net/)^52^. In brief, sequences >200-bp long with an average quality score >25 and no ambiguous characters were included in the analyses. Bacterial and archaeal phylotypes were identified using Uclust^53^ and assigned to OTUs based on 97% similarity. A representative sequence was chosen from each phylotype by selecting the most highly connected sequence^51^. All representative sequences were aligned by Py NAST^54^. The taxonomic identity (Supplementary Table 5) of each phylotype was determined using the Greengenes database (http://greengenes.lbl.gov/). Filtering of the sequences to remove erroneous, OTUs due to sequence errors and chimeras was conducted using the USEARCH tool in QIIME, version 1.8.0. Because we relied on filtering rather than denoising of the data, we also deleted singletons and set a threshold for a high-quality score (that is, 30) when running the command split_libraries.py, similar to approaches from other studies (for example, refs 51, 55, 56). Total sequence counts were normalized to 4,000 and 2,000 sequences per sample for bacterial and archaeal samples, respectively (Supplementary Fig. 12). AM fungal sequences were processed and analysed using Mothur v.1.30.1 (ref. 57). The fasta, quality and flow files were extracted from the standard flowgram format file of Roche, and were trimmed and de-noised (pdiffs=0, bdiffs=0, minflows=360, maxflows=550, maxhomop=8, minlength=230 and flip=T). Remaining sequences >230 bp in length^49^ were aligned to the reference SILVA database^58^. Sequences not aligning within the optimized alignment region were removed from the analysis with the screening function. The remaining sequencing were processed to reduce sequencing noise by a pre-clustering methodology^59^, and to remove chimeric sequences using UCHIME^60^. The OTUs were assigned by an average neighbour algorithm with a 0.03 dissimilarity cutoff, based on the sequences and/or OTUs obtained within the phylum of Glomeromycota. In spite of the specificity of the AM fungal primer, Alveolata, Metazoa, Viridiplantae, Stramenopiles, Dikarya and unclassified consensus taxonomy were detected; they were removed as environmental contaminants. AM fungal OTUs corresponding to unique AM virtual taxa were defined on the basis of a BLAST search of representative sequences from each OTU against the MaarjAM database^61^ (Supplementary Table 5; Supplementary Fig. 12).

### Soil fauna extraction and identification

We assessed soil faunal biodiversity by collecting and homogenizing three soil cores within each plot (3.5 cm in diameter, 0–15 cm in depth). Cores were returned to the lab and soil fauna were extracted using a modified Berlese–Tullgren apparatus^62^. To be more specific, soil samples for microarthopods who prefer dry environments were extracted through Tullgren funnels for 48 h (dry funnel method). Soil samples for nematodes and enchytraeids, who prefer wet environments, were wrapped in nylon cloth and extracted through Berlese funnels for 48 h (wet funnel method). They were counted, identified at order level, and preserved in 75% ethyl alcohol.

### Plant biomass measurement and identification

Before soil sampling, the plant communities were surveyed and harvested in each 1-m^2^ plot to measure standing aboveground biomass. The plant material was dried at 60 °C for ∼12 h and weighed. Then, we ground the aboveground plant material to a fine powder on a ball mill and analysed for plant nitrogen, and phosphorus using the techniques outlined for soils above. We estimated root biomass by collecting 4–8 soil cores per 1-m^2^ plot (0–5-cm deep, 7-cm diameter). A ratio of live:dead (56%)^63^ was used to calibrate root biomass. Plant species richness was assessed at three plots (1 × 1 m^2^) located 10-m away from the soil samples. We listed all vascular plant species for each of the 180 plots.

### Quantifying ecosystem multifunctionality

In total, we collected data that quantify key ecosystem functions and related variables: (1) aboveground biomass, (2) root biomass, (3) soil organic carbon, (4) soil nitrogen, (5) soil available nitrogen, (6) soil phosphorus, (7) plant nitrogen (nitrogen pools in aboveground biomass), and (8) plant phosphorus (phosphorus pools in aboveground biomass).

There are several ways to estimate the relationship between biodiversity and EMF^21^: the single functions approach, turnover approach, averaging approach and threshold approach. Because each approach has its strengths and weaknesses^21^, we used three distinct methods to calculate multifunctionality: single functions approach, an averaging approach, and a multiple thresholds approach. The relationships between EMF and biodiversity using the multiple thresholds approach were very similar to that obtained with the averaging approach (hereafter, EMF index), and hence we used the EMF index as described by Maestre *et al*.^9^ in the main text and in further analyses. We acknowledge that the EMF index, calculated from functions that are often correlated with one other (Supplementary Figs 13 and 14), does not provide a single best metric of EMF^21^. We use the EMF index for two reasons: (1) we are interested in biological or service-based outcomes^21^, and (2) this method is a straightforward and interpretable measure of a community's ability to sustain multiple functions simultaneously^9^. To obtain the EMF index for each site, we calculated the Z-scores for each of the eight ecosystem functions evaluated. The Z-scores for the measured variables were averaged to obtain an EMF for each site.

### Estimating biodiversity

We related EMF to biodiversity for each site, where biodiversity is the complete tally of all species at each sampled site. More specifically, plant biodiversity (species richness) is the number of plant species, bacterial and archaeal biodiversity is the number of OTUs, AM fungal richness is the number of virtual taxa, and faunal richness is the number of taxonomic units at the order level tallied at each site. We were conservative in our estimates and used a similarity of 97% (ref. 64) to delineate OTUs to represent soil bacteria and soil archaea species. We used virtual taxa for AM fungi, which are defined with bootstrap support and a sequence similarity of ≥97% (ref. 65). Note that we use the term ‘biodiversity' throughout, although the microbial and archaeal groups are phylotypes, AM fungal groups are virtual taxa, faunal groups are taxa at the order level. In addition, we used an approach of averaging standardized values of belowground biodiversity into a single index (soil biodiversity) as described by Wagg *et al*.^11^. An index was used from the average of all standardized biodiversity indices (bacterial OTUs, archaeal OTUs, faunal richness and AM fungal richness) to represent changes in soil biodiversity.

### Statistical analyses

The soil characteristics, ecosystem functions and above- and belowground biodiversity were assessed for a total of 180 plots. However, we use site means for the data analyses, except for the biodiversity assessments that were based on number of species or OTUs present in each site (*n*=60). First, we evaluated the relationships between biodiversity and each component of EMF (Supplementary Figs 3–8), as well as the relationships between biodiversity and EMF relationship (Fig. 1) using OLS regressions. Then, we evaluated the combined effects of above- and belowground biodiversity on EMF by fitting GLMs (Supplementary Table 2). And, we fitted partial linear regression as described in Quinn and Keough^66^ to identify the relative biodiversity effects of soil and plant biodiversity on EMF (Fig. 2). The partial linear regression allowed us to estimate how much of the variation in EMF can be exclusively attributed to soil and plant biodiversity. To further determine whether the observed effects of soil and plant biodiversity influenced EMF as much as abiotic factors, we conducted both regression and correlation analyses for seven predictors including MAP, MAT, soil moisture, soil pH, soil CaCO_3_, soil biodiversity and plant species richness (Supplementary Figs 9 and 10). Then, we fitted GLMs to investigate the biotic and abiotic effects on EMF (Table 1). To address multicollinearity, we first conducted a PCA using a correlation matrix with the seven predictors mentioned above. Seven components of this PCA were retained for further analyses. Two components that had eigenvalues >1 (PC1=1.871, PC2=1.120), explaining 68% variation in the data. We fitted two models of GLMs, one is a full model with the seven components, the other one is a reduced model with two components (PC1 and PC2) (Supplementary Fig. 11). Then, we used variables with VIF (variance inflation factor)<3 to select variables for the GLMs analyses (Table 1).

In addition, because the partial linear regression and the GLMs assume that the biodiversity and/or abiotic effects are additive, but not interactive, we further fitted a piecewise SEM (piecewise SEM)^67^ to infer relative importance of climate (MAP and MAT), soil (soil moisture and soil pH) and biodiversity (soil and plant) on EMF. Compared with the traditional variance covariance-based SEM, the piecewise SEM could (1) piece multiple separate (generalized) linear models together to a single causal network, (2) use Shipley's test of d-separation to test whether any paths are missing from the model and (3) use Akaike information criterion (AIC) to compare nested models and for small sample size (http://jonlefcheck.net/2014/07/06/piecewise-structural-equation-modeling-in-ecological-research/). We constructed the piecewise SEM based on the schematic diagram in the Supplementary Fig. 1. Because piecewise SEM is only for recursive models, we selected two models for the piecewise SEM; one assumed that plant species richness drives belowground biodiversity, the other one assumed that belowground biodiversity drives plant species richness (Fig. 3). We fitted the component models of the piecewise SEM as linear models. We reported the standardized coefficient for each path from each component models. Overall fit of the piecewise SEM was evaluated using Shipley's test of d-separation: Fisher's *C* statistic^68^ and AIC^69^ in the R package ‘piecewiseSEM' (https://github.com/jslefche/piecewiseSEM). MAP, soil pH, aboveground biomass, belowground biomass, soil organic carbon, soil nitrogen, soil available nitrogen, soil phosphorus, plant nitrogen and plant phosphorus were log-transformed. Soil moisture, soil CaCO_3_, bacterial, faunal, archaeal, AM fungal and plant biodiversity were sqrt-transformed. All statistical analyses were performed using R version 3.0.2 (R Foundation for Statistical Computing, Vienna, Austria, 2013).

## Additional information

**Accession codes:** The 454 pyrosequencing data set of soil bacteria are deposited in the DDBJ Sequence Read Archive (http://trace.ddbj.nig.ac.jp/DRASearch) with accession number: DRA001226, the 454 pyrosequencing data set of soil archaea are deposited in the European Nucleotide Archive (http://www.ebi.ac.uk/ena) with accession number: PRJEB8007, and the 454 pyrosequencing data set of soil AM fungi are deposited in the NCBI Bio Project Archive (http://www.ncbi.nlm.nih.gov/bioproject/269515) with accession number: PRJNA269515.

**How to cite this article:** Jing, X. *et al*. The links between ecosystem multifunctionality and above- and belowground biodiversity are mediated by climate. *Nat. Commun.* 6:8159 doi: 10.1038/ncomms9159 (2015).

## Supplementary Material

Supplementary Information Supplementary Figures 1-14, Supplementary Tables 1-5, Supplementary Note and Supplementary References

## Figures and Tables

**Table 1:** Summary of the general linear models (GLMs) for the effects of abiotic factors (soil moisture, soil pH and mean annual temperature) and biodiversity (plant species richness and soil biodiversity) on ecosystem multifunctionality (EMF).

Source	Estimate	SE	*t*-value	Significance *P*_r_ (>|*t*|)	MS	*F*-value	Significance *P*_r_ (>*F*)	VIF
*Multiple R*^*2*^ *0.86; residual SE 0.265 on 54 df*								
SM	1.492	0.138	10.818	< 0.001	17.17	243.753	< 0.001	1.286
pH	−1.111	0.529	−2.100	0.04038	1.82	25.833	< 0.001	1.466
Plant SR	0.178	0.063	2.832	0.00648	2.59	36.735	< 0.001	2.343
Soil biodiversity	0.307	0.103	2.995	0.00414	0.64	9.048	0.003988	1.939
MAT	0.037	0.013	2.820	0.00671	0.56	7.951	0.006705	1.081

df, degree of freedom; MS, mean square; MAT, mean annual temperature; pH, soil pH; plant SR, plant species richness; SE, standard errors; SM, soil moisture; VIF, variance inflation factor.

The best-fit model of the GLMs can be expressed as: EMF=0.982+1.492 × SM−1.111 × pH+0.178 × plant SR+0.307 × soil biodiversity+0.037 × MAT.

## References

[b1] Tilman D., Wedin D. & Knops J. Productivity and sustainability influenced by biodiversity in grassland ecosystems. Nature 379, 718–720 (1996).

[b2] Tilman D. . The influence of functional diversity and composition on ecosystem processes. Science 277, 1300–1302 (1997).

[b3] Hector A. . Plant diversity and productivity experiments in European grasslands. Science 286, 1123–1127 (1999). 10550043 10.1126/science.286.5442.1123

[b4] Tilman D. . Diversity and productivity in a long-term grassland experiment. Science 294, 843–845 (2001). 11679667 10.1126/science.1060391

[b5] Hillebrand H. & Matthiessen B. Biodiversity in a complex world: consolidation and progress in functional biodiversity research. Ecol. Lett. 12, 1405–1419 (2009). 19849711 10.1111/j.1461-0248.2009.01388.x

[b6] Kirwan L. . Evenness drives consistent diversity effects in intensive grassland systems across 28 European sites. J. Ecol. 95, 530–539 (2007).

[b7] Hector A. & Bagchi R. Biodiversity and ecosystem multifunctionality. Nature 448, 188–190 (2007). 17625564 10.1038/nature05947

[b8] Isbell F. . High plant diversity is needed to maintain ecosystem services. Nature 477, 199–202 (2011). 21832994 10.1038/nature10282

[b9] Maestre F. T. . Plant species richness and ecosystem multifunctionality in global drylands. Science 335, 214–218 (2012). 22246775 10.1126/science.1215442 PMC3558739

[b10] Gamfeldt L., Hillebrand H. & Jonsson P. R. Multiple functions increase the importance of biodiversity for overall ecosystem functioning. Ecology 89, 1223–1231 (2008). 18543617 10.1890/06-2091.1

[b11] Wagg C., Bender S. F., Widmer F. & van der Heijden M. G. A. Soil biodiversity and soil community composition determine ecosystem multifunctionality. Proc. Natl Acad. Sci. USA 111, 5266–5270 (2014). 24639507 10.1073/pnas.1320054111 PMC3986181

[b12] Lavelle P. Diversity of soil fauna and ecosystem function. Biol. Int. 33, 3–16 (1996).

[b13] van der Heijden M. G. A. . Mycorrhizal fungal diversity determines plant biodiversity, ecosystem variability and productivity. Nature 396, 69–72 (1998).

[b14] Zak D. R., Holmes W. E., White D. C., Peacock A. D. & Tilman D. Plant diversity, soil micrbial communities, and ecsystem function: are there any links? Ecology 84, 2042–2050 (2003).

[b15] van der Heijden M. G. A., Bardgett R. D. & van Straalen N. M. The unseen majority: soil microbes as drivers of plant diversity and productivity in terrestrial ecosystems. Ecol. Lett. 11, 296–310 (2008). 18047587 10.1111/j.1461-0248.2007.01139.x

[b16] Bardgett R. D. & Wardle D. A. Aboveground-Belowground Linkages: Biotic Interactions, Ecosystem Processes, and Global Change Oxford Univ. Press (2010).

[b17] Eisenhauer N., Reich P. B. & Isbell F. Decomposer diversity and identity influence plant diversity effects on ecosystem functioning. Ecology 93, 2227–2240 (2012). 23185884 10.1890/11-2266.1

[b18] Cardinale B. J. . Biodiversity loss and its impact on humanity. Nature 486, 59–67 (2012). 22678280 10.1038/nature11148

[b19] Darwin C. The Formation of Vegetable Mould, Through the Action of Worms, with Observations on Their Habits John Murray (1881).

[b20] Bardgett R. D. & van der Putten W. H. Belowground biodiversity and ecosystem functioning. Nature 515, 505–511 (2014). 25428498 10.1038/nature13855

[b21] Byrnes J. E. K. . Investigating the relationship between biodiversity and ecosystem multifunctionality: challenges and solutions. Methods Ecol. Evol. 5, 111–124 (2014).

[b22] Bowker M. A., Maestre F. T. & Mau R. L. Diversity and patch-size distributions of biological soil crusts regulate dryland ecosystem multifunctionality. Ecosystems 16, 923–933 (2013).

[b23] Wolters V. . Effects of global changes on above- and belowground biodiversity in terrestrial ecosystems: implications for ecosystem functioning. Bioscience 50, 1089–1098 (2000).

[b24] Wardle D. A. . Ecological linkages between aboveground and belowground biota. Science 304, 1629–1633 (2004). 15192218 10.1126/science.1094875

[b25] Pfisterer A. B. & Schmid B. Diversity-dependent production can decrease the stability of ecosystem functioning. Nature 416, 84–86 (2002). 11882897 10.1038/416084a

[b26] Ma W. . Environmental factors covary with plant diversity-productivity relationships among Chinese grassland sites. Glob. Ecol. Biogeogr. 19, 233–243 (2010).

[b27] Ulrich W. . Climate and soil attributes determine plant species turnover in global drylands. J. Biogeogr. 41, 2307–2319 (2014). 25914437 10.1111/jbi.12377 PMC4407967

[b28] Tedersoo L. . Global diversity and geography of soil fungi. Science 346, 1256688 (2014). 25430773 10.1126/science.1256688

[b29] Adler P. B. . Productivity is a poor predictor of plant species richness. Science 333, 1750–1753 (2011). 21940895 10.1126/science.1204498

[b30] Pasari J. R., Levi T., Zavaleta E. S. & Tilman D. Several scales of biodiversity affect ecosystem multifunctionality. Proc. Natl Acad. Sci. USA 100, 10219–10222 (2013). 23733963 10.1073/pnas.1220333110 PMC3690867

[b31] Balvanera P. . Quantifying the evidence for biodiversity effects on ecosystem functioning and services. Ecol. Lett. 9, 1146–1156 (2006). 16972878 10.1111/j.1461-0248.2006.00963.x

[b32] Scherber C. . Bottom-up effects of plant diversity on multitrophic interactions in a biodiversity experiment. Nature 468, 553–556 (2010). 20981010 10.1038/nature09492

[b33] Pringle R. M., Young T. P., Rubenstein D. I. & McCauley D. J. Herbivore-initiated interaction cascades and their modulation by productivity in an African savanna. Proc. Natl Acad. Sci. USA 104, 193–197 (2007). 17190823 10.1073/pnas.0609840104 PMC1765433

[b34] Liu Z., Liu G., Fu B. & Zheng X. Relationship between plant species diversity and soil microbial functional diversity along a longitudinal gradient in temperate grasslands of Hulunbeir, Inner Mongolia, China. Ecol. Res. 23, 511–518 (2007).

[b35] Torsvik V., Ovreas L. & Thingstad T. F. Prokaryotic diversity--magnitude, dynamics, and controlling factors. Science 296, 1064–1066 (2002). 12004116 10.1126/science.1071698

[b36] Smith S. E. & Read D. J. Mycorrhizal Symbiosis Academic Press (2010).

[b37] Leininger S. . Archaea predominate among ammonia-oxidizing prokaryotes in soils. Nature 442, 806–809 (2006). 16915287 10.1038/nature04983

[b38] Yang Y. . Soil carbon stock and its changes in northern China's grasslands from 1980s to 2000s. Glob. Change Biol. 16, 3036–3047 (2010).

[b39] Shi Y. . Field-based observations of regional-scale, temporal variation in net primary production in Tibetan alpine grasslands. Biogeosciences 10, 16843–16878 (2013).

[b40] Hooper D. . Effects of biodiversity on ecosystem functioning: a consensus of current knowledge. Ecol. Monogr. 75, 3–35 (2005).

[b41] Baumann F., He J.-S., Schmidt K., Kühn P. & Scholten T. Pedogenesis, permafrost, and soil moisture as controlling factors for soil nitrogen and carbon contents across the Tibetan Plateau. Glob. Change Biol. 15, 3001–3017 (2009).

[b42] Yang Y. . Significant soil acidification across northern China's grasslands during 1980s–2000s. Glob. Change Biol. 18, 2292–2300 (2012).

[b43] Fierer N. & Jackson R. B. The diversity and biogeography of soil bacterial communities. Proc. Natl Acad. Sci. USA 103, 626–631 (2006). 16407148 10.1073/pnas.0507535103 PMC1334650

[b44] Shi X. . Soil database of 1: 1,000,000 digital soil survey and reference system of the Chinese genetic soil classification system. Soil Surv. Horiz. 45, 129–136 (2004).

[b45] Chu H. . Soil bacterial diversity in the Arctic is not fundamentally different from that found in other biomes. Environ. Microbiol. 12, 2998–3006 (2010). 20561020 10.1111/j.1462-2920.2010.02277.x

[b46] Caporaso J. G. . Global patterns of 16S rRNA diversity at a depth of millions of sequences per sample. Proc. Natl Acad. Sci. USA 108, (Suppl 1): 4516–4522 (2011). 20534432 10.1073/pnas.1000080107 PMC3063599

[b47] Muyzer G., Teske A., Wirsen C. & Jannasch H. Phylogenetic relationships of Thiomicrospira species and their identification in deep-sea hydrothermal vent samples by denaturing gradient gel electrophoresis of 16S rDNA fragments. Arch. Microbiol. 164, 165–172 (1995). 7545384 10.1007/BF02529967

[b48] Yu Z., Garcia-Gonzalez R., Schanbacher F. L. & Morrison M. Evaluations of different hypervariable regions of archaeal 16S rRNA genes in profiling of methanogens denaturing by Archaea-specific PCR and gradient gel electrophoresis. Appl. Environ. Microbiol. 74, 889–893 (2008). 18083874 10.1128/AEM.00684-07 PMC2227698

[b49] Lumini E., Orgiazzi A., Borriello R., Bonfante P. & Bianciotto V. Disclosing arbuscular mycorrhizal fungal biodiversity in soil through a land-use gradient using a pyrosequencing approach. Environ. Microbiol. 12, 2165–2179 (2010). 21966911 10.1111/j.1462-2920.2009.02099.x

[b50] Sato K., Suyama Y., Saito M. & Sugawara K. A new primer for discrimination of arbuscular mycorrhizal fungi with polymerase chain reaction-denature gradient gel electrophoresis. Grassl. Sci. 51, 179–181 (2005).

[b51] Hamady M., Walker J. J., Harris J. K., Gold N. J. & Knight R. Error-correcting barcoded primers for pyrosequencing hundreds of samples in multiplex. Nat. Methods 5, 235–237 (2008). 18264105 10.1038/nmeth.1184 PMC3439997

[b52] Caporaso J. G. . QIIME allows analysis of high-throughput community sequencing data. Nat. Methods 7, 335–336 (2010). 20383131 10.1038/nmeth.f.303 PMC3156573

[b53] Edgar R. C. Search and clustering orders of magnitude faster than BLAST. Bioinformatics. 26, 2460–2461 (2010). 20709691 10.1093/bioinformatics/btq461

[b54] DeSantis T. Z. . NAST: a multiple sequence alignment server for comparative analysis of 16S rRNA genes. Nucleic Acids Res. 34, W394–W399 (2006). 16845035 10.1093/nar/gkl244 PMC1538769

[b55] Jones R. T. . A comprehensive survey of soil acidobacterial diversity using pyrosequencing and clone library analyses. ISME J. 3, 442–453 (2009). 19129864 10.1038/ismej.2008.127 PMC2997719

[b56] Lauber C. L., Hamady M., Knight R. & Fierer N. Pyrosequencing-based assessment of soil pH as a predictor of soil bacterial community structure at the continental scale. Appl. Environ. Microbiol. 75, 5111–5120 (2009). 19502440 10.1128/AEM.00335-09 PMC2725504

[b57] Schloss P. Introducing mothur: Open-source, platform-independent, community-293 supported software for describing and comparing microbial communities. Appl. Environ. Microbiol. 294, 7537–7541 (2009). 19801464 10.1128/AEM.01541-09 PMC2786419

[b58] Pruesse E. . SILVA: a comprehensive online resource for quality checked and aligned ribosomal RNA sequence data compatible with ARB. Nucleic Acids Res. 35, 7188–7196 (2007). 17947321 10.1093/nar/gkm864 PMC2175337

[b59] Huse S. M., Welch D. M., Morrison H. G. & Sogin M. L. Ironing out the wrinkles in the rare biosphere through improved OTU clustering. Environ. Microbiol. 12, 1889–1898 (2010). 20236171 10.1111/j.1462-2920.2010.02193.x PMC2909393

[b60] Edgar R. C., Haas B. J., Clemente J. C., Quince C. & Knight R. UCHIME improves sensitivity and speed of chimera detection. Bioinformatics. 27, 2194–2200 (2011). 21700674 10.1093/bioinformatics/btr381 PMC3150044

[b61] Öpik M. . The online database MaarjAM reveals global and ecosystemic distribution patterns in arbuscular mycorrhizal fungi (Glomeromycota). New Phytol. 188, 223–241 (2010). 20561207 10.1111/j.1469-8137.2010.03334.x

[b62] André H. M., Ducarme X. & Lebrun P. Soil biodiversity: myth, reality or conning? Oikos 96, 3–24 (2002).

[b63] Yang Y., Fang J., Ji C. & Han W. Above- and belowground biomass allocation in Tibetan grasslands. J. Veg. Sci. 20, 177–184 (2009).

[b64] Schloss P. D. & Handelsman J. Introducing DOTUR, a computer program for defining operational taxonomic units and estimating species richness. Appl. Environ. Microbiol. 71, 1501–1506 (2005). 15746353 10.1128/AEM.71.3.1501-1506.2005 PMC1065144

[b65] Öpik M., Metsis M., Daniell T. J., Zobel M. & Moora M. Large-scale parallel 454 sequencing reveals host ecological group specificity of arbuscular mycorrhizal fungi in a boreonemoral forest. New Phytol. 184, 424–437 (2009). 19558424 10.1111/j.1469-8137.2009.02920.x

[b66] Quinn G. P. & Keough M. J. Experimental Design and Data Analysis for Biologists Cambridge Univ. Press (2002).

[b67] Lefcheck J. S. & Duffy J. E. Multitrophic functional diversity predicts ecosystem functioning in experimental assemblages of estuarine consumers. PeerJ. 3, e1137 (2015). 27070016 10.1890/14-1977.1

[b68] Shipley B. Confirmatory path analysis in a generalized multilevel context. Ecology 90, 363–368 (2009). 19323220 10.1890/08-1034.1

[b69] Shipley B. The AIC model selection method applied to path analytic models compared using a d-separation test. Ecology 94, 560–564 (2013). 23687881 10.1890/12-0976.1

