# Supplementary material for: The links between ecosystem multifunctionality and above- and belowground biodiversity are mediated by climate
Source: Nat Commun. 2015 Sep 2;6:8159. doi: 10.1038/ncomms9159 (PMC4569729; doi:10.1038/ncomms9159)
Supplement: Supplementary Information — Supplementary Figures 1-14, Supplementary Tables 1-5, Supplementary Note and Supplementary References [file ncomms9159-s1.pdf]

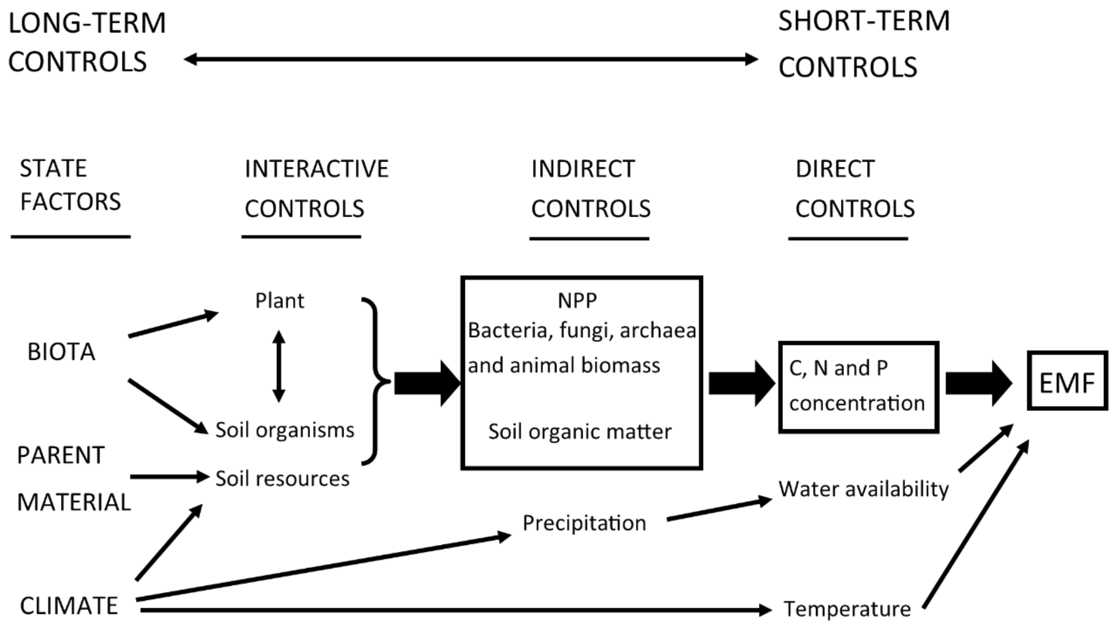

Supplementary Figure 1. Schematic diagram showing the major factors governing spatial variation in ecosystem multifunctionality (EMF), which related to C, N, and P cycling and storages. Those controls include state factors, interactive controls, indirect controls, and direct controls, which are the ultimate causes of ecosystem differences in EMF. The factors that account for most of the variation among ecosystems in EMF are C, N, and P concentration, water availability and temperature, which are ultimately determined by the interacting effects of soil organisms, soil resources, climate, and vegetation. The diagram was modified according to Chapin & Matson (2011) [1] and Maestre *et al.* (2012) [2].

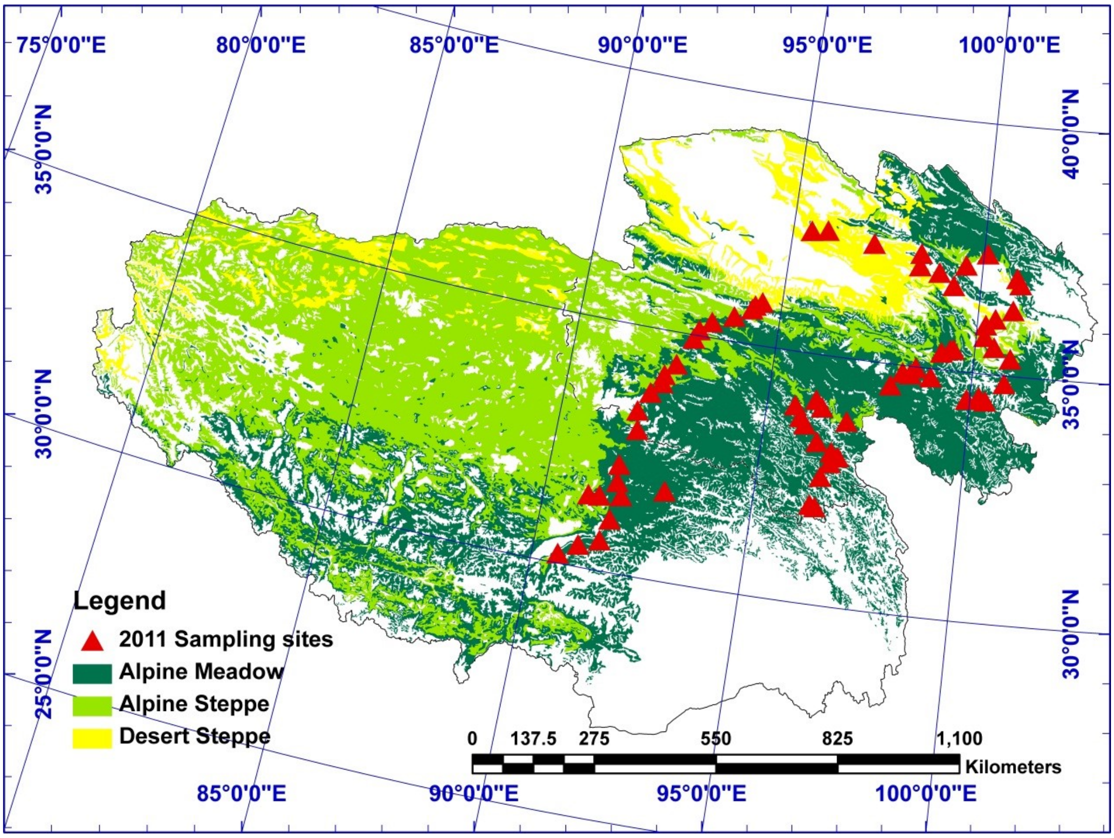

Supplementary Figure 2. Vegetation map of the Tibetan Plateau grasslands and location of sampling sites (1:1,000,000) [3].

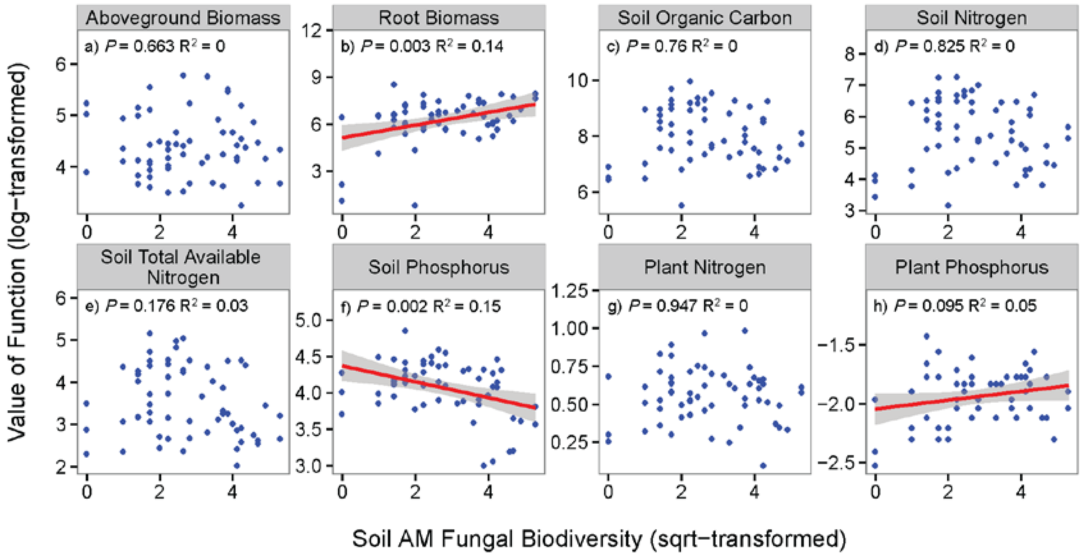

Supplementary Figure 3. Relationship between soil AMF fungal virtual taxon and each component of ecosystem multifunctionality. a) aboveground biomass, b) root biomass, c) soil organic carbon, d) soil nitrogen, e) soil total available nitrogen, f) soil phosphorus, g) nitrogen in aboveground biomass, h) phosphorus in aboveground biomass. Red lines are the fitted lines from OLS regressions. Shaded areas show the 95% CI of the fit.

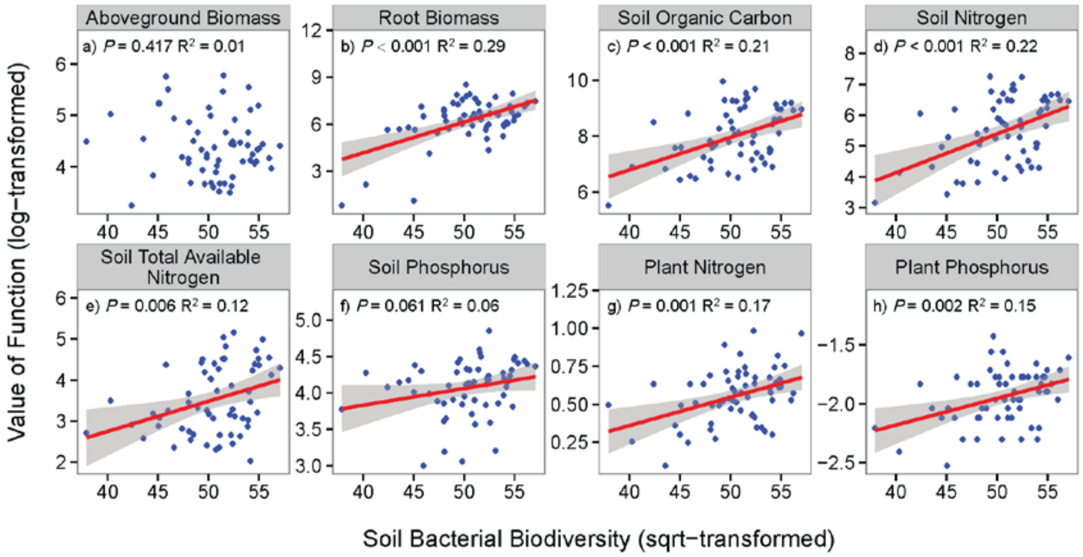

Supplementary Figure 4. Relationship between soil bacterial OTUs and each component of ecosystem multifunctionality. a) aboveground biomass, b) root biomass, c) soil organic carbon, d) soil nitrogen, e) soil total available nitrogen, f) soil phosphorus, g) nitrogen in aboveground biomass, h) phosphorus in aboveground biomass. Red lines are the fitted lines from OLS regressions. Shaded areas show the 95% CI of the fit.

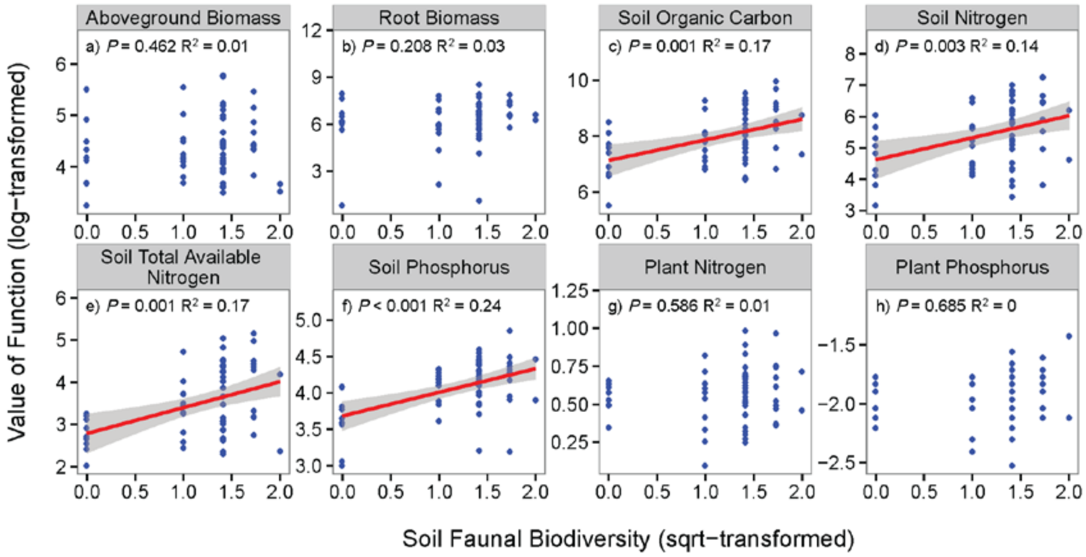

Supplementary Figure 5. Relationship between soil faunal richness and each component of ecosystem multifunctionality. a) aboveground biomass, b) root biomass, c) soil organic carbon, d) soil nitrogen, e) soil total available nitrogen, f) soil phosphorus, g) nitrogen in aboveground biomass, h) phosphorus in aboveground biomass. Red lines are the fitted lines from OLS regressions. Shaded areas show the 95% CI of the fit.

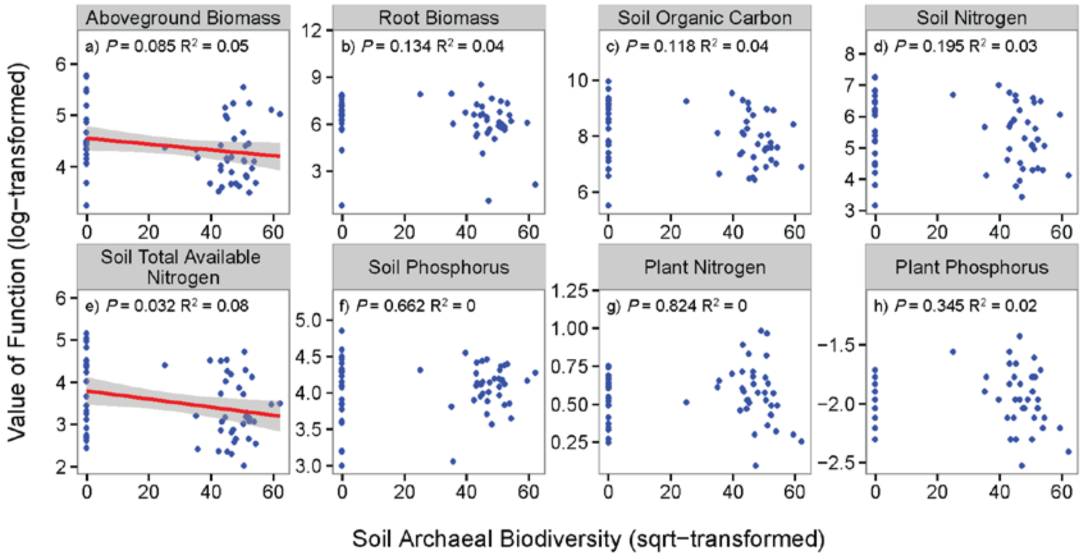

Supplementary Figure 6. Relationship between soil archaeal OTUs and each component of ecosystem multifunctionality. a) aboveground biomass, b) root biomass, c) soil organic carbon, d) soil nitrogen, e) soil total available nitrogen, f) soil phosphorus, g) nitrogen in aboveground biomass, h) phosphorus in aboveground biomass. Red lines are the fitted lines from OLS regressions. Shaded areas show the 95% CI of the fit.

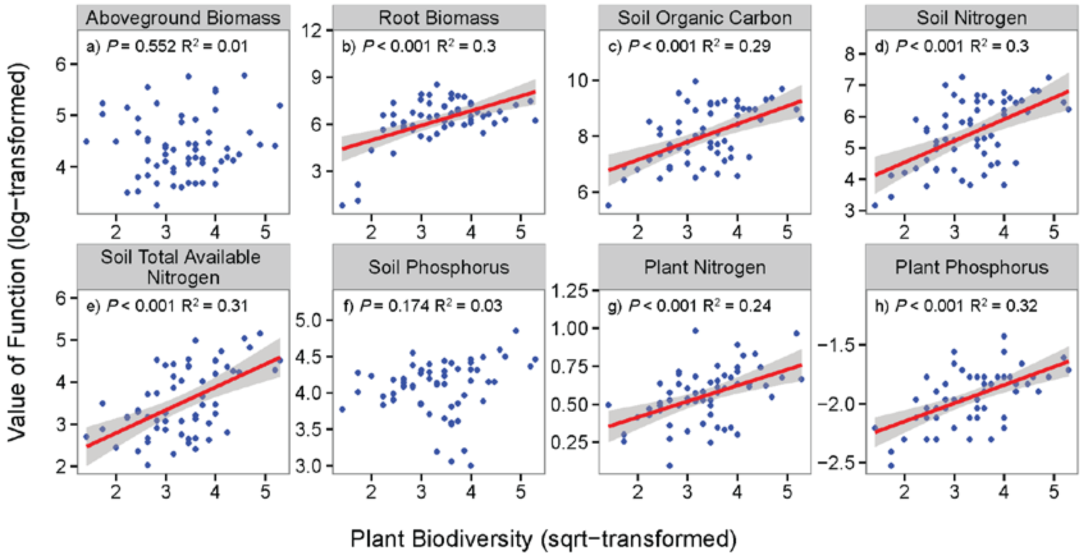

Supplementary Figure 7. Relationship between plant species richness and each component of ecosystem multifunctionality. a) aboveground biomass, b) root biomass, c) soil organic carbon, d) soil nitrogen, e) soil total available nitrogen, f) soil phosphorus, g) nitrogen in aboveground biomass, h) phosphorus in aboveground biomass. Red lines are the fitted lines from OLS regressions. Shaded areas show the 95% CI of the fit.

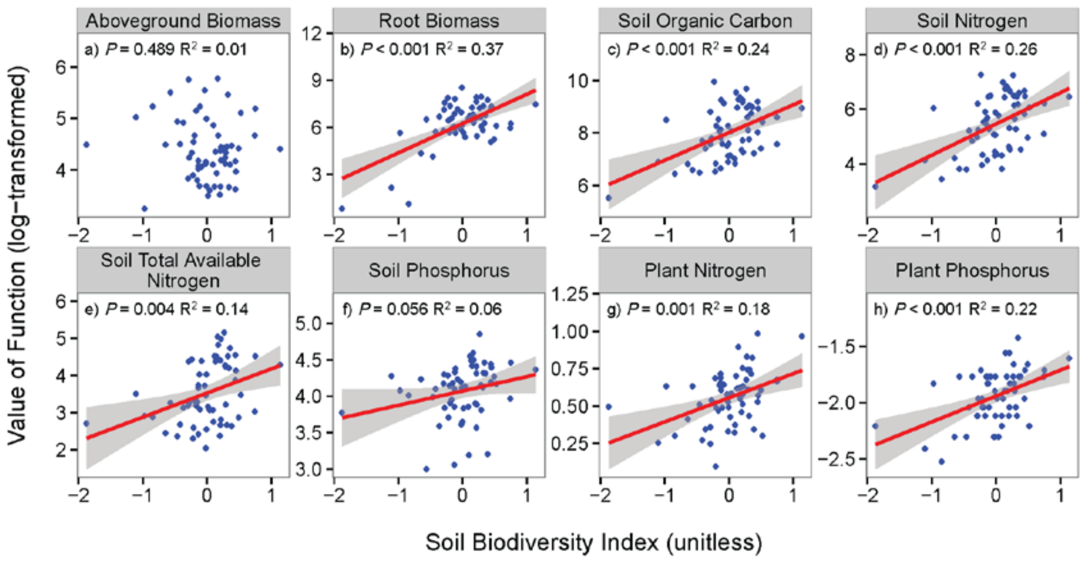

Supplementary Figure 8. Relationship between soil biodiversity and each component of ecosystem multifunctionality. a) aboveground biomass, b) root biomass, c) soil organic carbon, d) soil nitrogen, e) soil total available nitrogen, f) soil phosphorus, g) nitrogen in aboveground biomass, h) phosphorus in aboveground biomass. Red lines are the fitted lines from OLS regressions. Shaded areas show the 95% CI of the fit.

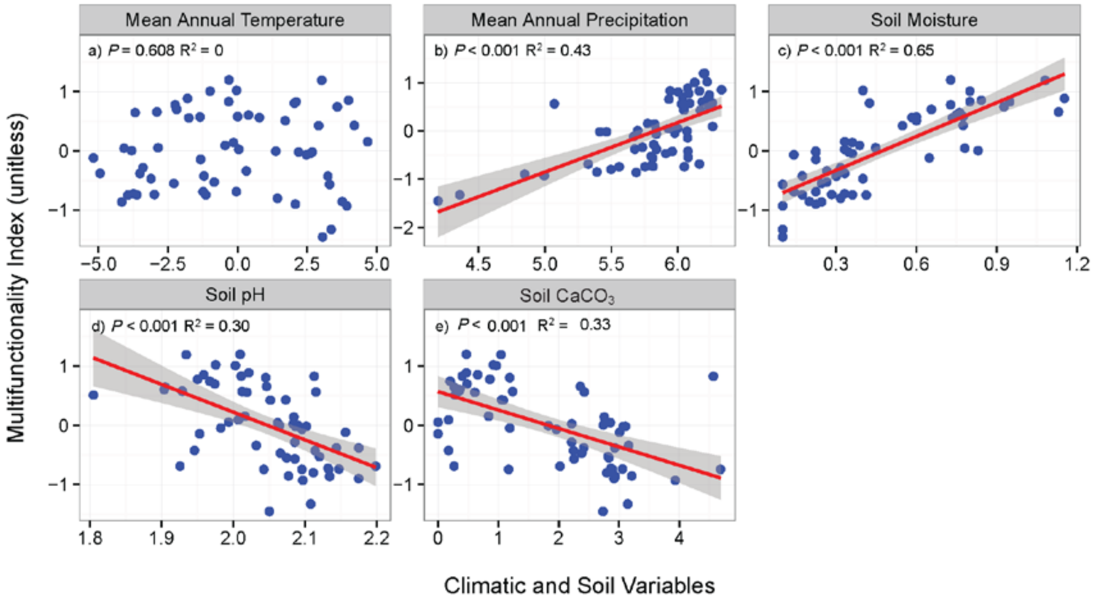

Supplementary Figure 9. Relationship between climatic and soil factors and ecosystem multifuctionality. a) mean annual temperature, b) mean annual precipitation, c) soil moisture, d) soil pH, e) soil $CaCO_3$. Red lines are the fitted lines from OLS regressions. Shaded areas show the 95% CI of the fit.

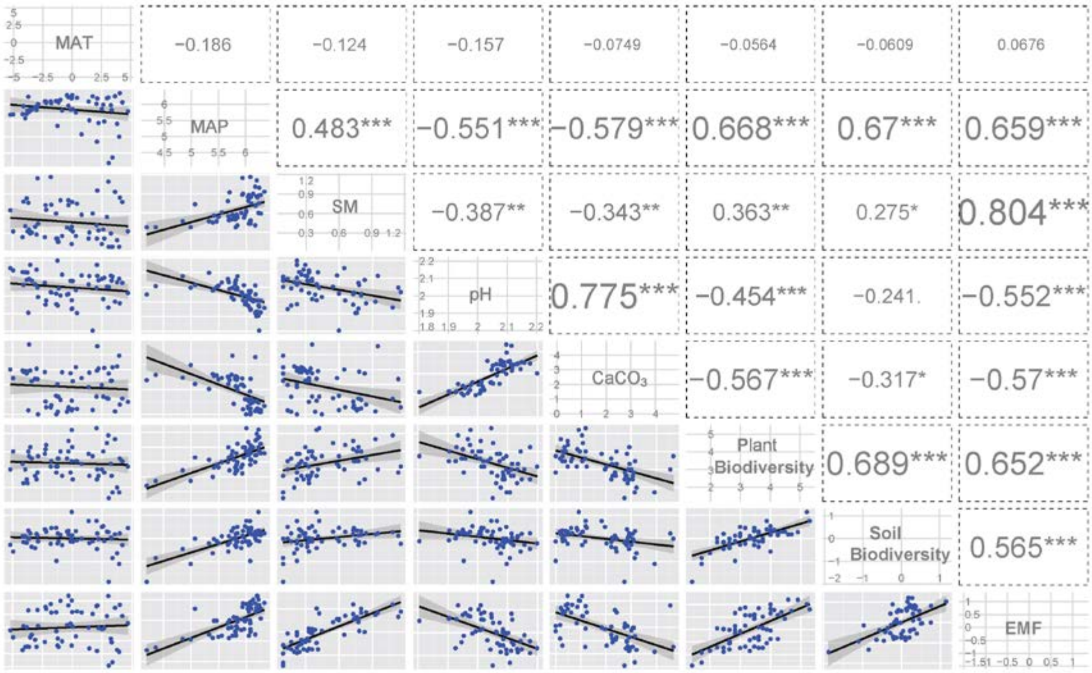

Supplementary Figure 10. Scatterplots matrices for the most important site variables (climate and soil) and ecosystem multifunctionality (EMF). MAT = mean annual temperature; MAP = mean annual precipitation; SM = soil moisture; pH = soil pH; $CaCO_3$ = soil $CaCO_3$. The upper triangular matrix shows the pairwise relationships among variables. *P*-value of the correlation coefficients are as follows: *** *P* <0.001, ** *P* <0.01, * *P* <0.05. The lower triangular matrix shows the linear relationships among variables. Shaded areas show the 95% CI of the fit.

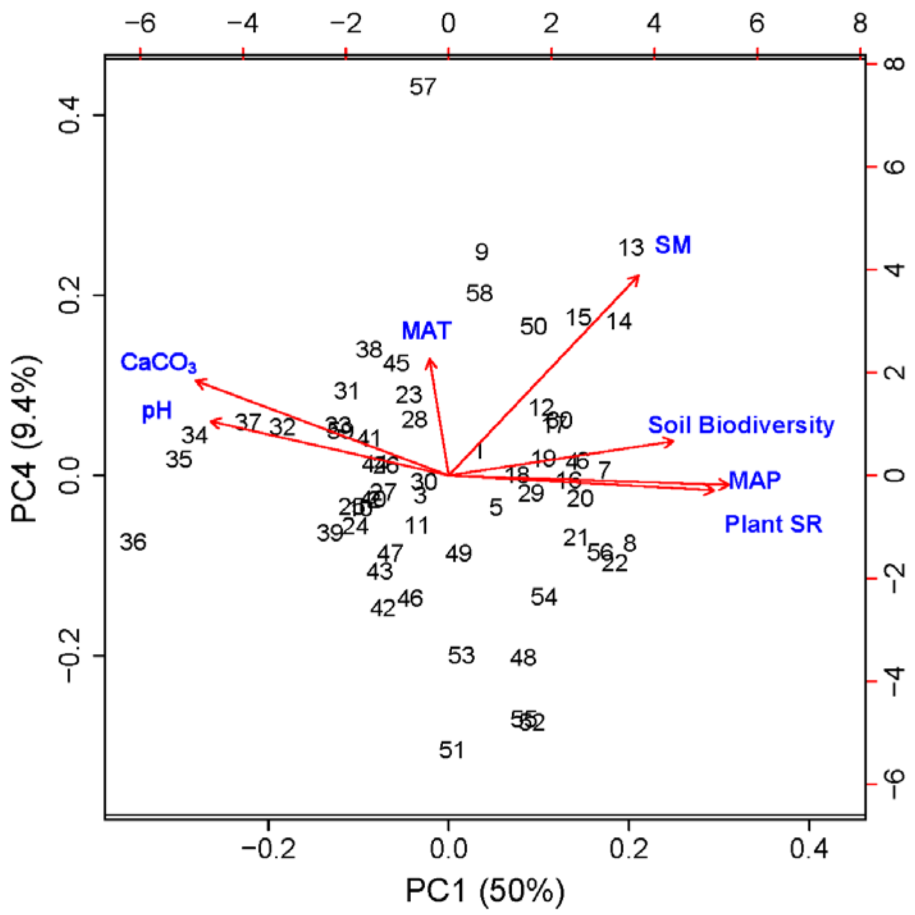

Supplementary Figure 11. Biplot derived from principal component analysis (PCA) including the principal components of PC1 and PC4 as well as the observations. The biotic and abiotic factors correlated with PC1 and PC4 are shown. MAP = mean auunal precipitation; MAT = mean annual temperature; SM = soil moisture; Plant SR = plant species richness. Different numbers refer to sites (n = 60).

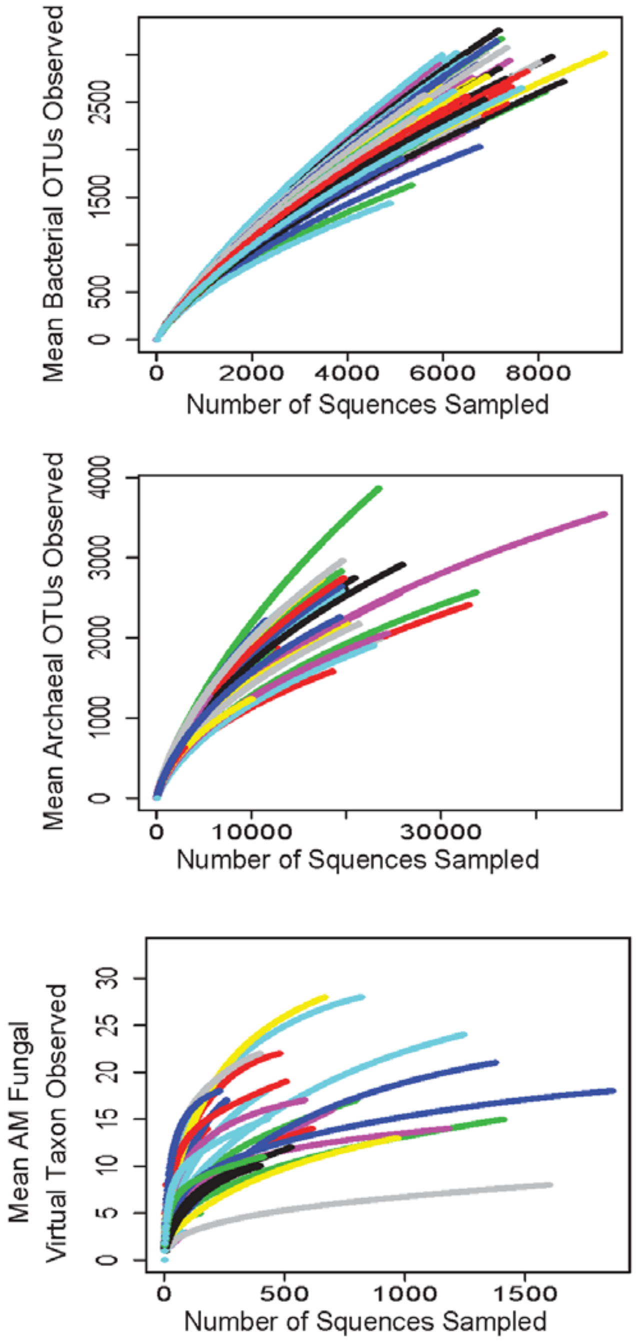

Supplementary Figure 12. Rarefaction curves showing read number and soil bacterial OTUs, archaeal OTUs, and AM fungal virtual taxon across all samples. Each sample is represented by different colours.

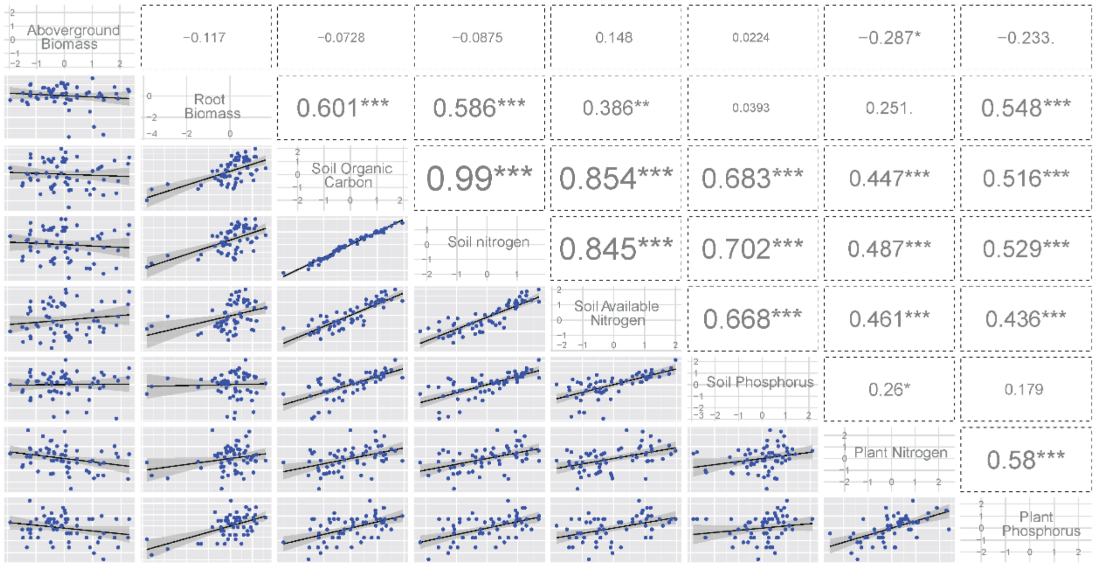

Supplementary Figure 13. Scatterplots matrices for the most important ecosystem functions and related variables used in this study. The upper triangular matrix shows the pairwise relationships among variables. *P*-value of the correlation coefficients are as follows: *** *P* <0.001, ** *P* <0.01, * *P* <0.05. The lower triangular matrix shows the linear relationships among variables. Shaded areas show the 95% CI of the fit.

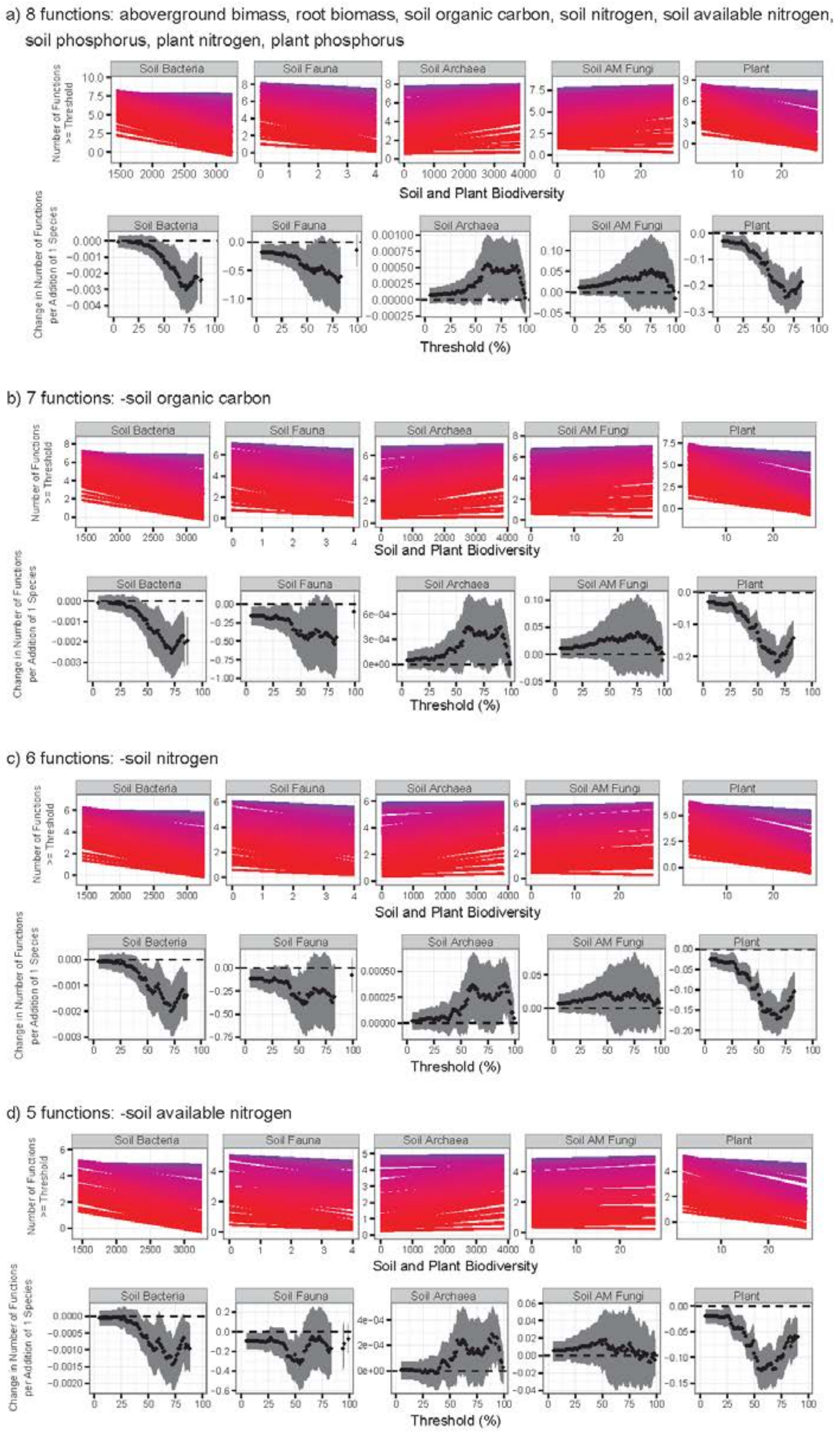

Supplementary Figure 14. Results of multiple threshold showing relationships between soil and plant biodiversity and ecosystem multifunctionality (EMF). The patterns of biodiversity effects on EMF did not change through removing one higher correlated function at each time.

Supplementary Table 1. Summary of site characteristics, climate variables, soil characteristics, ecosystem functions, and biodiversity of soil and plant in this study.

**Variables**	**Description**	**Units**
longitude	site characteristics	*°E*
latitude	site characteristics	*°N*
altitude	site characteristics	*m*
mean annual temperature	climate variables	*ºC*
mean annual precipitation	climate variables	*mm*
soil moisture	soil characteristics	*g/g*
soil pH (KCl)	soil characteristics	*unitless*
soil $CaCO_3$ density	soil characteristics	g/m^2
soil bulk density	soil characteristics	g/cm^3
soil organic carbon density	variables used to calculate EMF	g/m^2
soil nitrogen density	variables used to calculate EMF	g/m^2
soil phosphorus density	variables used to calculate EMF	g/m^2
total available nitrogen	variables used to calculate EMF; sum of ammonium, nitrate, and DON	*mg/kg*
aboveground biomass	variables used to calculate EMF	g/m^2
root biomass	variables used to calculate EMF	g/m^2
plant nitrogen	variables used to calculate EMF; total nitrogen in aboveground biomass	*%*
plant phosphorus	variables used to calculate EMF; total phosphorus in aboveground biomass	*%*
EMF	ecosystem multifucntionality, averaging of z scores of ecosystem functions	*unitless*
soil bacterial biodiversity	number of OTU	*integer*
soil faunal biodiversity	number of fauna at order level	*integer*
soil archaeal biodiversity	number of OTU	*integer*
soil AM fungal biodiversity	number of AM fungal virtual taxa	*integer*
plant biodiversity	plant species richness	*integer*
soil biodiversity	soil biodiversity index, averaging of z scores of soil biodiversity	*unitless*

Supplementary Table 2. Summary of the general linear models (GLMs) and ANOVA for the effects of soil and plant biodiversity on ecosystem multifunctionality (EMF). The regression model can be expressed as: EMF = -4.754e-17 + 0.315Soil Biodiversity + 0.336Plant Biodiversity.

Source	Estimate	SE	*t*-value	Significance Pr (>|*t*|)	MS	*F*-value	Significance Pr (>*F*)
Multiple R^2 0.45; residual SE 0.506 on 57 df							
Soil Biodiversity enter first							
Soil Biodiversity	0.315	0.194	1.624	0.109854	8.48	33.066	< 0.001
Plant Biodiversity	0.336	0.091	3.687	0.000508	3.49	13.591	0.0005083
Plant Biodiversity enter first							
Plant Biodiversity	0.336	0.091	3.687	0.000508	11.29	44.019	< 0.001
Soil Biodiversity	0.315	0.194	1.624	0.109854	0.68	2.638	0.1099

SE, standard errors; MS, mean square; df, degree of freedom.

Supplementary Table 3. Summary of the general linear models (GLMs) for the effects of PCA components on ecosystem multifunctionality (EMF). We derived seven components from the abiotic factors (mean annual temperature, mean annual precipitation, soil moisture, soil pH, soil $CaCO_3$), and biotic factors (plant species richness, soil biodiversity index) that mostly influenced EMF. The full model of GLMs with the seven components accounts for 86% variation in EMF. However, the best-fit model of GLMs shows that the components of PC1 and PC4 are the drivers of EMF, which accounting for 84% variation in EMF. The biotic and abiotic factors correlated with PC1 and PC4 are shown in the biplot (a summary map including the PC1 and PC4 as well as the observations) for principal components (Supplementary Figure S11).

Source	Estimate	SE	*t*-value	Significance Pr (>|*t*|)	Proportion of Variance
**Full model**					
Multiple R^2 0.86; residual SE 0.264 on 53 df					
PC1	0.2927	0.0184	15.923	<0.001	0.5002
PC2	0.0089	0.0310	0.286	0.7760	0.1759
PC3	0.0691	0.0364	1.899	0.0631	0.1277
PC4	0.3477	0.0424	8.199	<0.001	0.0940
PC5	0.1023	0.0603	1.696	0.0958	0.0465
PC6	0.0780	0.0773	1.010	0.3173	0.0283
PC7	-0.0570	0.0784	-0.728	0.4702	0.02751
**Best-fit model**					
Multiple R^2 0.84; residual SE 0.271 on 57 df					
PC1	0.2927	0.0189	15.505	<0.001	0.5002
PC4	0.3477	0.0436	7.984	<0.001	0.0940

SE, standard errors; df, degree of freedom; Proportion of variance shows the proportional variance of each component from the PCA analysis.

Supplementary Table 4. Description of the geographic, climatic, and soil variables by this study sites (n = 60). MAT, Mean annual temperature; MAP, mean annual precipitation; SM, soil moisture; SSTC, soil organic carbon stocks; SSTN, soil total nitrogen stocks; TAN, soil total available nitrogen; SSTP, soil total phosphorous stocks; SCaCO3, soil $CaCO_3$ stocks; soil bulk density (SBD).

	min	max	range	median	mean	SE.mean	std.dev
Longitude (°E)	90.62	101.02	10.40	96.38	96.17	0.43	3.36
Latitude (°N)	30.20	37.48	7.28	34.71	34.44	0.25	1.97
Altitude (m)	2918	5228	2310	4239	4064	77	599
MAT (°C)	-5.18	4.67	9.85	-0.23	-0.17	0.36	2.75
MAP (mm yr^{-1})	66.45	560.17	493.72	378.90	365.39	14.78	114.50
SM (W/W %)	0.01	1.33	1.32	0.13	0.30	0.04	0.33
Soil pH	6.08	9.01	2.93	7.87	7.77	0.08	0.60
SSOC (g/m^2)	247.68	21026.34	20778.66	3093.48	4732.55	568.21	4401.32
SSTN (g/m^2)	23.33	1413.33	1390.00	261.67	377.17	44.00	340.86
TAN (mg/kg)	7.59	173.34	165.75	27.22	47.37	5.14	39.81
SSTP (g/m^2)	20.15	127.68	107.53	62.99	62.33	2.62	20.33
SCaCO3 (g/m^2)	0.00	12469.18	12469.18	2147.11	2639.16	352.34	2729.18
SBD (g/m^3)	0.43	1.50	1.07	1.04	1.00	0.03	0.27

Supplementary Table 5. Taxonomic assignment, absolute, and relative sequence frequency of the top 25 abundant bacterial OTUs, archaeal OTUs, and AM fungal VTX (virtual taxon).

	Order	Family	Genus	No. of OTU	No. of reads	% in total reads
**Bacterial OTU id**						
11283	Actinomycetales	Geodermatophilaceae	*Blastococcus*	180	8366	2.18
17580	Rhizobiales	unclasified	*unclasified*	173	8094	2.11
8107	Bacillales	unclasified	*unclasified*	172	5979	1.56
35167	ActinobacteriaGp4	unclasified	*unclasified*	180	4580	1.19
33318	Rhodospirillales	Rhodospirillaceae	*Skermanella*	172	3759	0.98
41641	ActinobacteriaGp4	unclasified	*unclasified*	160	3681	0.96
57397	Rhizobiales	unclasified	*unclasified*	172	3459	0.90
20597	unclasified	unclasified	*unclasified*	157	3354	0.87
334	Actinomycetales	Nocardioidaceae	*Nocardioides*	169	2261	0.59
25218	ActinobacteriaGp4	unclasified	*unclasified*	167	2201	0.57
4727	unclasified	unclasified	*unclasified*	136	2157	0.56
1312	unclasified	unclasified	*unclasified*	160	2137	0.56
19607	Rhizobiales	Phyllobacteriaceae	*unclasified*	176	2097	0.55
49990	unclasified	unclasified	*unclasified*	157	1908	0.50
55934	Rhizobiales	unclasified	*unclasified*	159	1880	0.49
821	Actinomycetales	unclasified	*unclasified*	151	1855	0.48
7029	Rhizobiales	Hyphomicrobiaceae	*unclasified*	167	1849	0.48
53217	Burkholderiales	Oxalobacteraceae	*Massilia*	167	1813	0.47
52553	ActinobacteriaGp4	unclasified	*unclasified*	167	1809	0.47
22261	Solirubrobacterales	unclasified	*unclasified*	170	1686	0.44
2658	Rhodospirillales	Rhodospirillaceae	*Skermanella*	154	1673	0.44
4842	Rhizobiales	unclasified	*unclasified*	161	1654	0.43
40215	Rhizobiales	unclasified	*unclasified*	125	1602	0.42
60800	Rhodospirillales	Acetobacteraceae	*Roseomonas*	159	1410	0.37
39552	Actinomycetales	Nocardioidaceae	*Nocardioides*	147	1299	0.34
**Archaeal OTU id**						
15722	Nitrososphaerales	Nitrososphaeraceae	*Nitrososphaera*	92	96398	14.11
934	Nitrososphaerales	Nitrososphaeraceae	*Nitrososphaera*	94	75265	11.02
17961	Nitrososphaerales	Nitrososphaeraceae	*Nitrososphaera*	93	42959	6.29
4265	Nitrososphaerales	Nitrososphaeraceae	*Nitrososphaera*	93	33540	4.91
6420	Nitrososphaerales	Nitrososphaeraceae	*Nitrososphaera*	94	31296	4.58
8126	Nitrososphaerales	Nitrososphaeraceae	*Nitrososphaera*	91	21635	3.17
5518	Nitrososphaerales	Nitrososphaeraceae	*Nitrososphaera*	88	11442	1.67
9088	Nitrososphaerales	Nitrososphaeraceae	*Nitrososphaera*	82	9166	1.34
5874	Nitrososphaerales	Nitrososphaeraceae	*Nitrososphaera*	87	6766	0.99
8243	Nitrososphaerales	Nitrososphaeraceae	*Nitrososphaera*	90	6731	0.99
18308	Nitrososphaerales	Nitrososphaeraceae	*Nitrososphaera*	78	5741	0.84
11666	Nitrososphaerales	Nitrososphaeraceae	*Nitrososphaera*	91	5620	0.82
12	Nitrososphaerales	Nitrososphaeraceae	*Nitrososphaera*	89	4659	0.68

3252	Nitrososphaerales	Nitrososphaeraceae	*Nitrososphaera*	88	4395	0.64
2653	Nitrososphaerales	Nitrososphaeraceae	*Nitrososphaera*	75	3410	0.50
12859	Nitrososphaerales	Nitrososphaeraceae	*Nitrososphaera*	90	2998	0.44
4575	Nitrososphaerales	Nitrososphaeraceae	*Nitrososphaera*	69	2890	0.42
9014	Nitrososphaerales	Nitrososphaeraceae	*Nitrososphaera*	89	2859	0.42
7743	Methanomicrobiales	unclasified	*unclasified*	7	2757	0.40
6366	Nitrososphaerales	Nitrososphaeraceae	*Nitrososphaera*	77	2564	0.38
1809	Halobacteriales	Halobacteriaceae	*unclasified*	4	2281	0.33
15987	Nitrososphaerales	Nitrososphaeraceae	*Nitrososphaera*	88	2268	0.33
6416	Nitrososphaerales	Nitrososphaeraceae	*Nitrososphaera*	84	2071	0.30
11572	Nitrososphaerales	Nitrososphaeraceae	*Nitrososphaera*	87	1946	0.28
16102	Nitrososphaerales	Nitrososphaeraceae	*Nitrososphaera*	89	1924	0.28
**AM Fungal virtual taxon**						
VTX00062	Diversisporales	Diversisporaceae	*Diversispora*	72	6996	36.89
VTX00325	Glomerales	Glomeraceae	*Glomus*	28	2878	15.18
VTX00167	Glomerales	Glomeraceae	*Glomus*	41	1832	9.66
VTX00054	Diversisporales	Diversisporaceae	*Diversispora*	50	1533	8.08
VTX00067	Glomerales	Glomeraceae	*Glomus*	17	1157	6.10
VTX00380	Diversisporales	Diversisporaceae	*Diversispora*	32	796	4.20
VTX00193	Glomerales	Claroideoglomeraceae	*Claroideoglomus*	70	678	3.58
VTX00085	Glomerales	Glomeraceae	*Glomus*	21	471	2.48
VTX00130	Glomerales	Glomeraceae	*Glomus*	51	321	1.69
VTX00027	Diversisporales	Acaulosporaceae	*Acaulospora*	3	254	1.34
VTX00064	Glomerales	Glomeraceae	*Glomus*	35	219	1.15
VTX00347	Diversisporales	Diversisporaceae	*Diversispora*	21	207	1.09
VTX00295	Glomerales	Glomeraceae	*Glomus*	26	201	1.06
VTX00247	Glomerales	Glomeraceae	*Glomus*	19	187	0.99
VTX00065	Glomerales	Glomeraceae	*Glomus*	28	172	0.91
VTX00319	Glomerales	Glomeraceae	*Glomus*	10	97	0.51
VTX00293	Glomerales	Glomeraceae	*Glomus*	9	92	0.49
VTX00165	Glomerales	Glomeraceae	*Glomus*	20	89	0.47
VTX00143	Glomerales	Glomeraceae	*Glomus*	26	77	0.41
VTX00231	Diversisporales	Acaulosporaceae	*Acaulospora*	7	73	0.38
VTX00188	Glomerales	Glomeraceae	*Glomus*	8	68	0.36
VTX00166	Glomerales	Glomeraceae	*Glomus*	25	49	0.26
VTX00030	Diversisporales	Acaulosporaceae	*Acaulospora*	11	48	0.25
VTX00177	Glomerales	Glomeraceae	*Glomus*	13	48	0.25
VTX00214	Glomerales	Glomeraceae	*Glomus*	6	46	0.24

Supplementary Note 1

Assessment of soil moisture: field sampling and rationale

1) Soil samples for soil moisture analysis were packed in polyethylene bags, immediately stored in portable refrigerator in the field, and then stored in the lab at -20°C. We measured soil moisture after no more than one week in the lab. We acknowledge that there are some shortcomings to the methodology. However, the protocol for soil moisture measurement has been used for decades on the Tibetan Plateau, it has been an efficient method and appears to be representative of soil characteristics and climatic conditions on the Tibetan Plateau [4, 5, 6, 7, 8, 9, 10]. We realize that other approaches are used in other systems, but in this system, this is the most appropriate way to measure soil moisture.

2) The Tibetan alpine grasslands have a unique growing-season climate that is dominated by summer monsoons, and are usually warm and wet. More than 85% of the annual precipitation occurs during the growing season (Wang et al. 2014). Meanwhile, because of bad weather and inaccessibility of the sites on the Tibetan Plateau, most of the previous samplings were conducted only once [4, 5, 6, 7, 8, 9, 10], and continuous field monitoring for the measurement of soil moisture is exceedingly rare. However, one of the studies from the Tibetan Plateau shows that the seasonal variation in soil moisture is relatively stable [11]. Therefore, we believe that soil moisture used in our study represents an indicator of the differences in available soil water throughout the climatic gradient studied.

3) Soil moisture used in this study is relatively more accurate than precipitation, because the dataset of mean annual precipitation was compiled by interpolating data of monthly precipitation records (1951–2010) from 716 climate stations across China (http://cdc.cma.gov.cn). Meanwhile, the moisture at the topsoil (5-cm depth) is "*more susceptible to strong radiation, diurnal temperature differences and accentuated precipitation events characterizing the special climate conditions on the Tibetan Plateau*" [5]. Therefore, soil moisture could be a stronger predictor of climatic conditions on the Tibetan Plateau.

Supplementary References

1. Chapin III FS, Matson PPA. *Principles of terrestrial ecosystem ecology*. Springer-Verlag (2011).

2. Maestre FT*, et al.* Plant species richness and ecosystem multifunctionality in global drylands. *Science* **335**, 214-218 (2012).

3. EBVMC Editorial Board of Vegetation Map of China CAS. *Vegetation atlas of China*. Science Press, Beijing (2001).

4. Yang Y*, et al.* Storage, patterns and controls of soil organic carbon in the Tibetan grasslands. *Global Change Biol.* **14**, 1592-1599 (2008).

5. Baumann F, He J-S, Schmidt K, Kühn P, Scholten T. Pedogenesis, permafrost, and soil moisture as controlling factors for soil nitrogen and carbon contents across the Tibetan Plateau. *Global Change Biol.* **15**, 3001-3017 (2009).

6. Ma W*, et al.* Environmental factors covary with plant diversity-productivity relationships among Chinese grassland sites. *Global Ecol. Biogeogr.* **19**, 233-243 (2010).

7. Shi Y*, et al.* Organic and inorganic carbon in the topsoil of the Mongolian and Tibetan grasslands: pattern, control and implications. *Biogeosciences* **9**, 2287-2299 (2012).

8. Geng Y*, et al.* Soil respiration in Tibetan alpine grasslands: belowground biomass and soil moisture, but not soil temperature, best explain the large-scale patterns. *PloS One* **7**, e34968 (2012).

9. Dorfer C, Kuhn P, Baumann F, He JS, Scholten T. Soil organic carbon pools and stocks in permafrost-affected soils on the tibetan plateau. *PLoS One* **8**, e57024 (2013).

10. Baumann F, Schmidt K, Dörfer C, He J-S, Scholten T, Kühn P. Pedogenesis, permafrost, substrate and topography: Plot and landscape scale interrelations of weathering processes on the central-eastern Tibetan Plateau. *Geoderma* **226**, 300-316 (2014).

11. Yu J, Liu X, Luo T, Zhang L. Seasonal variations of soil temperature and moisture at the upper limit of alpine meadow in North-facing slope of the Nianqingtanggula Mountains. *Acta Geographica Sinica* **67**, 1246-1254 (2012).
